# Assessing residential PM_2.5_ concentrations and infiltration factors with high spatiotemporal resolution using crowdsourced sensors

**DOI:** 10.1073/pnas.2308832120

**Published:** 2023-12-04

**Authors:** David M. Lunderberg, Yutong Liang, Brett C. Singer, Joshua S. Apte, William W. Nazaroff, Allen H. Goldstein

**Affiliations:** ^a^Department of Environmental Science, Policy, and Management, University of California, Berkeley, CA 94720; ^b^Department of Chemistry, University of California, Berkeley, CA 94720; ^c^College of Engineering, School of Chemical and Biomolecular Engineering, Georgia Institute of Technology, Atlanta, GA 30332; ^d^Indoor Environment Group, Energy Technologies Area, Lawrence Berkeley National Laboratory, Berkeley, CA 94720; ^e^Department of Civil and Environmental Engineering, University of California, Berkeley, CA 94720; ^f^Environmental Health Sciences Division, School of Public Health, University of California, Berkeley, CA 94720

**Keywords:** indoor air, PM_2.5_, infiltration, source apportionment, exposure

## Abstract

Building conditions, outdoor climate, and human behavior influence residential concentrations of fine particulate matter (PM_2.5_). To study PM_2.5_ spatiotemporal variability in residences, we acquired paired indoor and outdoor PM_2.5_ measurements at 3,977 residences across the United States totaling >10,000 monitor-years of time-resolved data (10-min resolution) from the PurpleAir network. Time-series analysis and statistical modeling apportioned residential PM_2.5_ concentrations to outdoor sources (median residential contribution = 52% of total, coefficient of variation = 69%), episodic indoor emission events such as cooking (28%, CV = 210%) and persistent indoor sources (20%, CV = 112%). Residences in the temperate marine climate zone experienced higher infiltration factors, consistent with expectations for more time with open windows in milder climates. Likewise, for all climate zones, infiltration factors were highest in summer and lowest in winter, decreasing by approximately half in most climate zones. Large outdoor–indoor temperature differences were associated with lower infiltration factors, suggesting particle losses from active filtration occurred during heating and cooling. Absolute contributions from both outdoor and indoor sources increased during wildfire events. Infiltration factors decreased during periods of high outdoor PM_2.5_, such as during wildfires, reducing potential exposures from outdoor-origin particles but increasing potential exposures to indoor-origin particles. Time-of-day analysis reveals that episodic emission events are most frequent during mealtimes as well as on holidays (Thanksgiving and Christmas), indicating that cooking-related activities are a strong episodic emission source of indoor PM_2.5_ in monitored residences.

## Significance

Until recently, residential studies of indoor fine particulate matter (PM_2.5_) have been limited to small numbers of homes observed for short periods of time. Advances in low-cost sensors and the advent of crowdsourced data collection have enabled thousands of homes to be studied over extended periods. To understand PM_2.5_ spatiotemporal variability across the residential building stock, this study uses more than 10,000 monitor-years of PM_2.5_ data acquired in or near 3,977 US residences. We quantify how indoor and outdoor sources contribute to residential PM_2.5_ by time of day, time of year, and climate. We find substantial variability related to occupant behavior, outdoor climate, and building conditions. By understanding such drivers, occupants and building designers can take steps to reduce residential exposure to PM_2.5_.

Exposure to fine particulate matter (PM_2.5_; particles smaller than 2.5 μm in aerodynamic diameter) is one of the leading risk factors for mortality, globally and in the United States (1, 2). The primary location of human PM_2.5_ exposure is indoors, where Western populations spend ~90% of their time (3–5). Indoor PM_2.5_ originates from both indoor and outdoor sources. Outdoor PM_2.5_ penetrates and persists indoors with partial and variable loss, owing to features such as building design and construction (envelope tightness), building operation (window use, filtration, and mechanical ventilation), and environmental conditions (outdoor wind speed and outdoor–indoor temperature difference) (6). These features vary among buildings and are also subject to substantial diurnal, seasonal, and geographic variability. The influence of such variability on indoor air pollution concentrations and pollution exposure is poorly understood. Despite their prominent influence on air pollution exposure, indoor environmental conditions are often excluded from public health studies and risk assessment models (7).

Indoor PM_2.5_ emission events can be short and produce sharp transient increases in indoor concentrations (8). The largest indoor emission events originate from cooking and (historically common) tobacco smoking, during which indoor PM_2.5_ concentrations may attain hundreds of μg m^−3^ (9, 10). Indoor sources also include particle resuspension (9, 11) and shedding (12); candle and incense use (13, 14); airborne chemistry (15–17); and condensation of indoor semivolatile gases onto outdoor particles (18, 19). The contributions of various sources across residences are not understood, in part due to the historical sparseness of extended residential PM_2.5_ datasets.

Considering that infiltration factors vary substantially by particle size, time of day, and time of year (6, 20), apportioning indoor PM_2.5_ concentrations between indoor and outdoor sources often requires simplifying assumptions (21). Time-integrated models, such as random component superposition (RCS) (22), are a computationally simple means to estimate infiltration factors, but these models often inaccurately treat the infiltration factor as a constant value. Use of dynamic models or infiltration surrogates can yield improved results, but these methods are challenging to apply at scale (21). Applying source-attribution methods to Western residences has yielded estimates that approximately 50 to 80% of residential indoor PM_2.5_ originates from outdoor PM_2.5_ that penetrates indoors and remains suspended (20, 23–29).

The recent expansion in the use of low-cost particle sensors via the PurpleAir sensor network has enabled investigations of indoor PM_2.5_ in hundreds to thousands of buildings (29–34), a scale much larger than previously possible. The Plantower particle sensors used by PurpleAir monitors agree well with regulatory monitoring stations, especially after applying correction factors to reduce negative sensor biases at low concentrations and positive sensor biases at high concentrations (35–38). Nevertheless, moderate biases and limitations still exist, related to heterogeneity in atmospheric conditions, particle size, and particle composition (33, 35, 36, 38). Attempts to apportion indoor PM_2.5_ into specific source categories have yet to differentiate between episodic and nonepisodic source categories and, more generally, to identify how source strengths vary across the building stock.

Here, using data from the PurpleAir network, we report an analysis of 562 million measurements of PM_2.5_ (at 10-min time resolution) inside and outside of ~4,000 residences across the United States, cumulatively representing >10,000 monitor-years of data. This multiyear analysis differentiates between episodic and persistent indoor sources, links PurpleAir monitors to specific residential buildings and uses building information in analysis. We report outdoor and indoor concentrations ( $C_{o}$ and $C_{i}$ , respectively), indoor concentrations of outdoor and indoor origin ( $C_{io}$ and $C_{ii}$ , respectively), and indoor concentrations from indoor episodic sources and indoor persistent sources ( $C_{iie}$ and $C_{iip}$ , respectively) at residences, and examine how these parameters change by time of day, time of year, and geographic location. We use these findings to interpret how key factors, such as building characteristics and human behavior, may be influencing PM_2.5_ concentrations in US residences.

## Results and Discussion

### Overview.

**Fig. 1.**
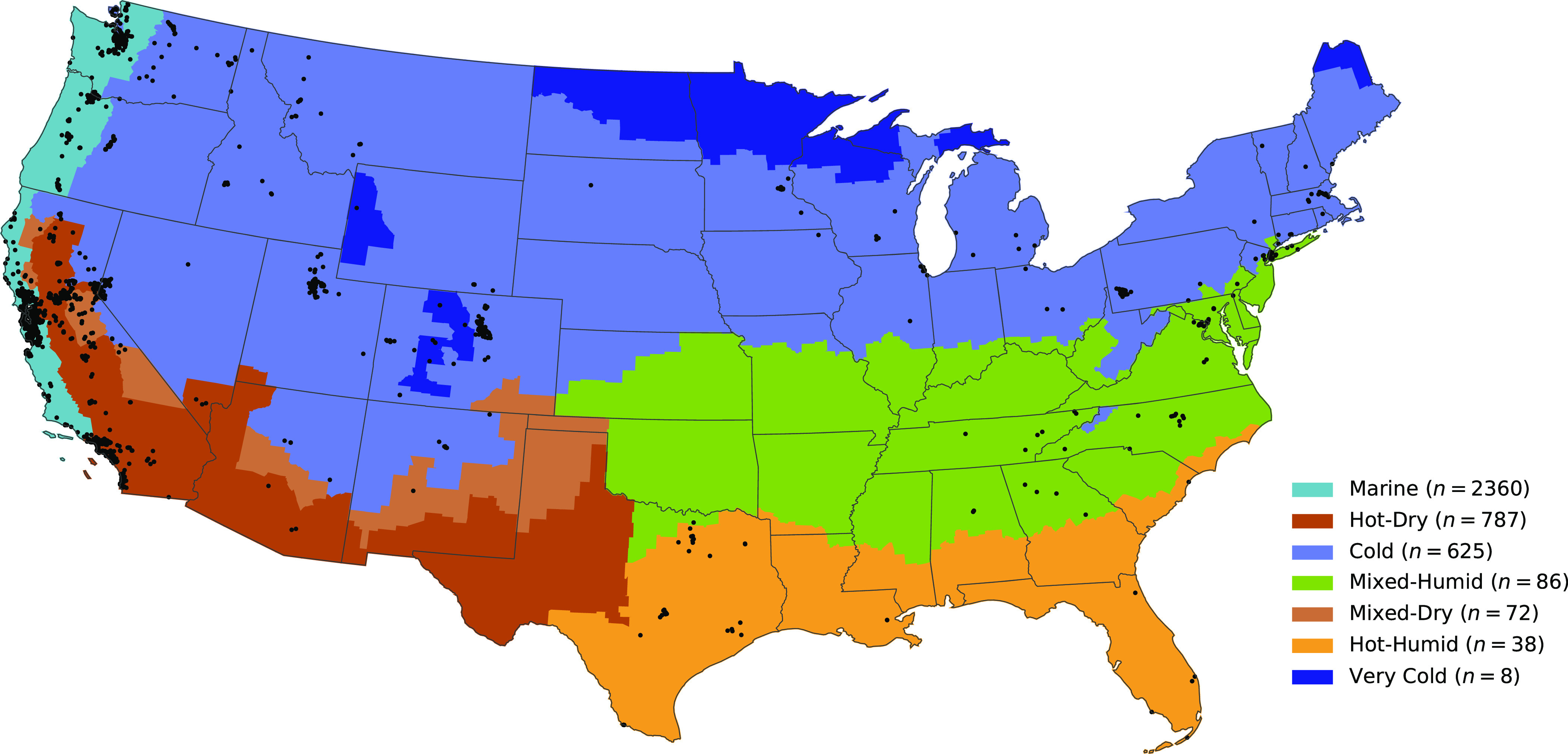
A map of climate zone designations used by the Department of Energy’s Building America program and selected residential monitors (black points) that passed quality assurance checks for the contiguous United States.

**Fig. 2.**
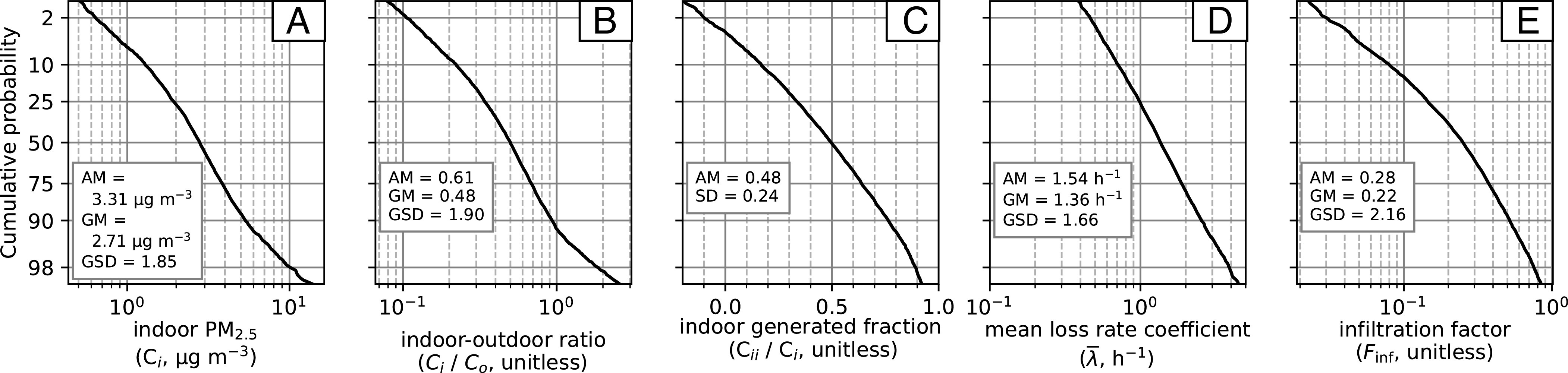
Distributions of indoor PM_2.5_ concentrations, indoor–outdoor ratios, PM_2.5_ fractions of indoor origin, particle loss-rate coefficients, and infiltration factors are displayed as cumulative probability plots in panels *A*–*E*), respectively, for 3,977 residential indoor–outdoor data pairs. The *x*-axis is linearly scaled in panel (*C*) and log-scaled in the remaining panels. With the given probability scale, normal (panel *C*) and log-normal (remaining panels) distributions would lie along a straight line. The arithmetic mean (AM), SD, geometric mean (GM), and geometric SD (GSD) are also reported, where relevant.

Using 3,977 paired indoor ( $C_{i}$   ) and outdoor ( $C_{o}$   ) concentration time series from US residences, we deconstructed the indoor concentrations into three additive terms reflecting different source characteristics: outdoor origin ( $C_{io}$   ), episodic indoor origin ( $C_{iie}$   ), and persistent (nonepisodic) indoor origin ( $C_{iip})$   . For each monitored residence, we also calculated first-order particle loss-rate coefficients ( $\lambda_{tot}$   ) associated with episodic emission events and infiltration factors ( $F_{inf})$   that quantify the indoor/outdoor concentration relationship. Data were obtained and analyzed for residences in 39 states in the contiguous US with the largest number of residences monitored in the West Coast states of California (*n* = 2,924), Washington (*n* = 259), and Oregon (*n* = 162). Most residences were in the Marine (*n* = 2,360), Cold (*n* = 625), or Hot-Dry (*n* = 787) climate zones (as defined by the Department of Energy Residential Buildings Program), with the remainder distributed among four other climate zones (“Other,” *n* = 205). Geographic locations of monitors can be found in Fig. 1 and *SI Appendix*, Table S1. Key summary statistics for the totality of the dataset are reported in Table 1 and Fig. 2 with stratifications across climate zone and season provided in *SI Appendix*, Tables S2–S5. Observed indoor and outdoor PM_2.5_ concentrations were 3.31 ± 2.96 μg m^−3^ and 5.99 ± 2.10 μg m^−3^, respectively (mean ± SD of residences). Time-averaged indoor concentrations were lower than outdoor concentrations in 92% of residences; the mean and median indoor–outdoor ratios by residence were 0.61 and 0.50, respectively. Infiltration factors, that is, the fraction of outdoor PM_2.5_ that penetrates indoors and remains suspended, were lower than reported in many prior studies, with the mean and median residence having values of 0.28 and 0.25, respectively. Indoor concentrations had roughly equal contributions from indoor (median = 50%) and outdoor (median = 50%) sources. Indoor sources were separated into indoor episodic sources (median = 55% of indoor contributions), probably dominated by cooking-related events, and indoor persistent sources (median = 45% of indoor contributions).

**Table 1.** Key summary statistics and major measurement results for monitors in residences linked to average values from all outdoor monitors within 5 km^*^

		Time-series analysis				Modified RCS analysis			Traditional RCS analysis		Indoor generated fractions		
		${\bar{\lambda }}_{tot}$	$C_{i}$	$C_{iie}$	$C_{o}$	$C_{io}$	$C_{iip}$	$F_{inf}$	$C_{ii}$	$F_{inf}$	$\frac{C_{iie}}{C_{i}}$	$\frac{C_{iip}}{C_{i}}$	$\frac{C_{iip}+C_{iie}}{C_{i}}$
	**Units**	h^−1^	μg m^−3^	μg m^−3^	μg m^−3^	μg m^−3^	μg m^−3^	None	μg m^−3^	None	None	None	None
Total	10th quantile	0.71	1.29	0.15	4.25	0.46	−0.08	0.08	0.27	0.08	0.09	−0.03	0.17
*n* = 3,977	**Median**	**1.36**	**2.79**	**0.62**	**5.59**	**1.39**	**0.52**	**0.25**	**1.19**	**0.26**	**0.24**	**0.20**	**0.50**
	90th quantile	2.54	5.34	2.31	8.38	2.92	1.15	0.52	3.18	0.53	0.55	0.44	0.76
	**Mean**	**1.54**	**3.31**	**1.19**	**5.99**	**1.60**	**0.52**	**0.28**	**1.70**	**0.29**	**0.28**	**0.20**	**0.48**
	SD	0.84	2.96	2.50	2.10	1.11	0.58	0.18	2.44	0.23	0.18	0.20	0.24
Marine	10th quantile	0.74	1.37	0.14	4.50	0.54	−0.05	0.09	0.28	0.10	0.08	−0.02	0.17
*n* = 2,360	**Median**	**1.37**	**2.78**	**0.55**	**5.41**	**1.46**	**0.54**	**0.27**	**1.14**	**0.27**	**0.21**	**0.20**	**0.48**
	90th quantile	2.58	4.83	2.03	6.93	2.77	1.1	0.51	2.87	0.52	0.48	0.44	0.72
	**Mean**	**1.56**	**3.12**	**0.98**	**5.78**	**1.61**	**0.52**	**0.29**	**1.52**	**0.29**	**0.25**	**0.20**	**0.46**
	SD	0.85	2.18	1.70	1.86	1.00	0.53	0.17	1.82	0.18	0.16	0.19	0.22
Hot-Dry	10th quantile	0.69	1.37	0.18	5.11	0.46	0.01	0.07	0.37	0.07	0.09	0	0.21
*n* = 787	**Median**	**1.35**	**3.02**	**0.64**	**6.76**	**1.47**	**0.67**	**0.22**	**1.34**	**0.23**	**0.22**	**0.23**	**0.51**
	90th quantile	2.50	5.67	2.24	9.25	3.16	1.42	0.49	3.36	0.49	0.50	0.46	0.75
	**Mean**	**1.51**	**3.56**	**1.15**	**7.00**	**1.72**	**0.68**	**0.26**	**1.89**	**0.26**	**0.26**	**0.23**	**0.50**
	SD	0.83	2.66	2.04	2.12	1.20	0.62	0.17	2.57	0.20	0.17	0.19	0.22
Cold	10th quantile	0.70	1.10	0.18	2.67	0.30	−0.33	0.06	0.12	0.05	0.13	−0.10	0.15
*n* = 625	**Median**	**1.36**	**2.53**	**0.87**	**4.93**	**1.00**	**0.34**	**0.22**	**1.23**	**0.21**	**0.39**	**0.14**	**0.60**
	90th quantile	2.59	6.89	3.37	8.41	2.94	1.00	0.56	4.38	0.58	0.69	0.39	0.85
	**Mean**	**1.54**	**3.43**	**1.69**	**5.43**	**1.41**	**0.33**	**0.27**	**2.07**	**0.27**	**0.40**	**0.13**	**0.53**
	SD	0.78	3.39	2.84	2.52	1.28	0.63	0.21	3.27	0.27	0.21	0.21	0.29
Other	10th quantile	0.60	0.97	0.15	3.49	0.42	−0.22	0.06	0.02	0.07	0.10	−0.11	0.09
*n* = 205	**Median**	**1.27**	**2.93**	**0.64**	**6.27**	**1.27**	**0.41**	**0.24**	**1.07**	**0.24**	**0.26**	**0.15**	**0.50**
	90th quantile	2.42	6.89	3.69	8.49	3.19	1.33	0.55	4.13	0.63	0.61	0.46	0.83
	**Mean**	**1.45**	**4.24**	**2.10**	**6.17**	**1.63**	**0.47**	**0.28**	**2.01**	**0.33**	**0.32**	**0.17**	**0.47**
	SD	0.90	7.20	6.76	2.03	1.28	0.66	0.21	4.43	0.54	0.20	0.23	0.28

^*^Concentration values ( $C_{i}$ , $C_{iie}$ , $C_{o}$ , $C_{io}$ , $C_{iip}$ , and $C_{ii}$  ) are reported in units of μg m^−3^. The infiltration factor, $F_{inf}$ , and the fractions of indoor PM_2.5_ of indoor episodic origin, indoor persistent origin, and total indoor origin, that is $C_{iie}$ /$C_{i}$ , $C_{iip}$/$C_{i}$ , and ${(C}_{iie}+C_{iip})$/$C_{i}$ , are unitless. The mean of well-behaved particle loss-rate coefficients at a residence ( ${\bar{\lambda }}_{tot}$ ) is reported in units of h^−1^. Bold formatted rows are used to guide the eye across the data table.

### Source Apportionment and Spatial Variation.

Source apportionment was conducted via a combination of time-series analysis and statistical modeling. Time-series analysis entailed identifying and integrating episodic concentration peaks that are indicative of indoor activities such as cooking and cleaning (*SI Appendix*, Fig. S1). The time-averaged concentration of episodically generated PM_2.5_ across residences ranged from 0.15 μg m^−3^ to 2.31 μg m^−3^ (10th–90th percentiles) with a median $C_{iie}$ value of 0.62 μg m^−3^. These contributions were highest in the Cold climate zone (median $C_{iie}$ value of 0.87 μg m^−3^) where the median episodically generated fraction of indoor PM_2.5_ across residences was 0.39, as opposed to 0.22 in the Hot-Dry climate zone, 0.21 in the Marine climate zone, and 0.26 in other climate zones (Table 1). The mean indoor-generated fraction contributed by episodic sources was 0.28. This finding can be compared to a recent report attributing 22% of daily PM_2.5_ exposures in homes to cooking events (39).

After generating an indoor time series from which all episodic peaks were removed, a modified form of random component superposition analysis was applied, yielding the apparent indoor concentration from persistent sources as the intercept of the regression line. These concentrations may be interpreted as originating from indoor sources that are continuously contributing to indoor PM_2.5_ (e.g., via oxidative chemistry forming secondary organic aerosols indoors or condensation of indoor semivolatile gases onto particles of outdoor origin) or from indoor sources that are either so frequent or small that they do not generate a sharp episodic peak (e.g., from resuspension or shedding from low-intensity occupant movement). Past studies have assumed that most indoor sources are episodic and that all indoor source influence can be removed by censoring episodic events in a time series (24, 27, 28). In contrast, indoor PM_2.5_ concentrations attributed to persistent indoor sources were found to be similar to episodically generated indoor PM_2.5_ concentrations, with mean concentrations across residences ranging from −0.08 μg m^−3^ to 1.15 μg m^−3^ (10th–90th percentiles) and a median $C_{iip}$ value of 0.52 μg m^−3^. (Negative concentrations yielded by modified RCS analysis are nonphysical artifacts from the statistical and regression models and may be interpreted as effectively zero.) Contributions from persistent indoor sources were highest in the Hot-Dry climate zone (median $C_{iip}$ = 0.66 μg m^−3^) where the median fraction of indoor PM_2.5_ attributed to persistent indoor emissions was 0.23 and lowest in the Cold climate zone (median $C_{iip}$ = 0.34 μg m^−3^) where the median fraction was 0.14.

Adding the contributions to indoor concentrations from persistent and episodic indoor emission yields the total indoor concentration of indoor origin ( $C_{ii})$   . We observed in all climate zones that indoor and outdoor source contributions to indoor concentrations were roughly equal, albeit with substantial variation among residences. The indoor-generated fraction varied from 0.17 to 0.76 (10th–90th percentiles) with a median of 0.50. Median indoor contributions were slightly larger in the Hot-Dry climate zone owing to their greater episodic contributions. These findings are consistent with prior studies that did not separate contributions between episodic and persistent sources (20, 25, 27, 29). The indoor concentration of outdoor origin can be estimated by mass balance or by applying the infiltration factor obtained from modified RCS analysis. Infiltration factors varied from 0.08 to 0.52 (10th–90th percentiles) with median $F_{inf}$ of 0.25. Accordingly, indoor concentrations of outdoor origin varied from 0.46 μg m^−3^ to 2.92 μg m^−3^ (10th–90th percentiles) with median $C_{io}$ of 1.39 μg m^−3^.

**Fig. 3.**
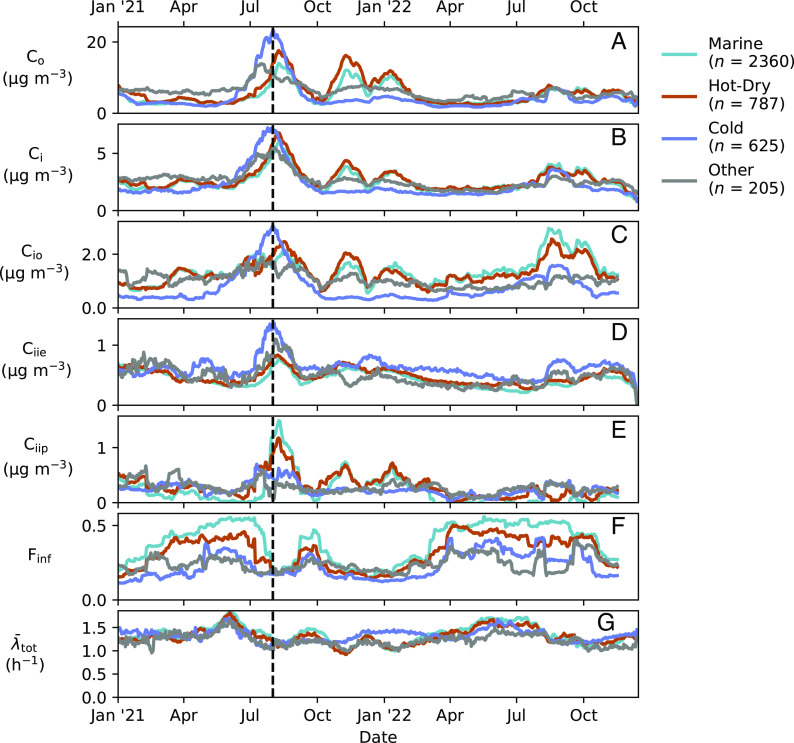
Time series of (*A*) outdoor PM_2.5_ ( $C_{o}$ , μg m^−3^), (*B*) indoor PM_2.5_ ( $C_{i}$ , μg m^−3^), (*C*) indoor PM_2.5_ of outdoor origin ( $C_{io}$ , μg m^−3^), (*D*) indoor PM_2.5_ originating from indoor episodic emissions ( $C_{iie}$ , μg m^−3^), (*E*) indoor PM_2.5_ originating from indoor persistent sources ( $C_{iip}$ , μg m^−3^), (*F*) infiltration factor ( $F_{inf}$ , unitless), and (*G*) mean particle loss-rate coefficient ( ${\bar{\lambda }}_{tot}$ , h^−1^). The data presented for each time series are the median values across all indoor–outdoor measurement pairs over a rolling 30-d window as grouped by climate zone. The vertical hatched line on 1 August 2021 marks a period of wildfires in the western United States.

While distinct trends across climate zones were clearly identifiable (Fig. 3) and variability among outdoor concentrations was low across the full residential dataset, remarkable heterogeneity was observed in key indoor parameters. The coefficient of variation in infiltrated ambient PM_2.5_ (69%) was nearly twice as high as that of ambient PM_2.5_ (CV of *C*_o_ = 35%). Variability in indoor-generated PM_2.5_ was 3–6 times as high as that of ambient PM_2.5_, with coefficients of variation of $C{}_{iip}$ and $C{}_{iie}$ equal to 111% and 211%, respectively. Even after stratifying the dataset by climate zone, most parameters displayed comparably large coefficients of variation (±30%). Differences within climate zones were often greater than differences between climate zones. Apart from differences between the Marine and Hot-Dry climate zones in median $C_{o}$ , the interquartile range within a climate zone was greater than the difference in median values between climate zones for every reported parameter in all climate zones. The climate zone grouping used here does not differentiate between the dry western United States (MT to NM and westward) and the higher humidity regions to the east. To explore whether observed variability may be attributed to differences in geography or humidity, we present key results for more detailed climate zone stratifications in *SI Appendix*, Table S6. Substantial heterogeneity remains within each of the more detailed climate zone stratifications. Understanding the key drivers of this variability may present opportunities for better characterizing residential exposures and for mitigating high exposures.

While outdoor and indoor PM_2.5_ concentrations generally increased with increasing population density, as expected, the highest values were observed in the least densely populated and most densely populated regions (*SI Appendix*, Table S7). We speculate that higher PM_2.5_ concentrations in regions with low population density may be attributed to the higher prevalence of residential wood-energy consumption in rural areas (40). Mean indoor PM_2.5_ concentrations in the least densely populated regions (<100 persons/km^2^) were 3.5 μg m^−3^, decreasing to 3.1 μg m^−3^ in the next least densely populated region (100–500 persons/km^2^). Mean indoor concentrations then increased with increasing population density, reaching 4.0 μg m^−3^ in the most densely populated region (>4,000 persons/km^2^). While no apparent trends were observed between mean infiltration factors and population density, mean particle loss rates were 7% higher in regions with population density greater than 1,000 persons/km^2^ than regions with population density lower than 1,000 persons/km^2^.

The indoor concentration attributed to persistent indoor sources, as inferred from the modified RCS model, requires qualifications during interpretation due to potential biases arising from concentration-dependent phase-change phenomena. We discuss these potential biases in *SI Appendix*.

### Temporal Trends and Infiltration Factors.

Substantial seasonal variability in PM_2.5_ origin and concentration was observed across climate zones (Fig. 3 and *SI Appendix*, Fig. S2). A striking feature is the influence of outdoor wildfires in 2021. These wildfire events produced high outdoor PM_2.5_ concentrations that penetrated indoors as observed in the Hot-Dry, Cold, and Marine climate zones across the western states. During a period of major wildfires in August 2021, monthly median outdoor PM_2.5_ concentrations increased to more than 20 μg m^−3^. Indoor concentrations increased by about 4 μg m^−3^ as outdoor PM_2.5_ penetrated indoors. As reported previously (33, 34) and as also inferred in this work, occupant actions attenuate PM_2.5_ infiltration. During periods of high outdoor air pollution, we observed decreased infiltration factors; these findings are consistent with protective actions advised by public health agencies of closing windows to reduce infiltration and using active air filtration, as available. While the lower infiltration factors resulted in lower indoor concentrations of outdoor-origin PM_2.5_ during the large wildfire events in 2021, indoor PM_2.5_ concentrations attributable to both indoor episodic and indoor persistent sources increased.

Separate from wildfire events, seasonal trends were observed in infiltration factors and particle loss-rate coefficients, with infiltration factors being highest in the summer and lowest in the winter across all climate zones (Fig. 3*F*). In the Marine and Hot-Dry climate zones, this decline approached 50%. Similarly, particle loss-rate coefficients were generally highest in summer and lowest in winter (Fig. 3*G*), plausibly due to enhanced ventilation rates during temperate summertime periods when windows are expected to be more frequently open. Two observations support this inference. First, infiltration factors were higher in more temperate climate zones, especially in spring and summer. Median infiltration factors in the Marine climate zone were sometimes up to double the corresponding values in the Cold climate zone. Infiltration factors for the Hot-Dry climate zone, where use of air conditioning is common, especially during afternoon summer heat, were between those of the Cold and Marine climate zones. This observation suggests that infiltration factors are coupled to building operation as influenced by outdoor temperature. Second, loss-rate coefficients in the Cold climate zone were markedly greater than those of any other climate zone during the winter season (Fig. 3*G* and *SI Appendix*, Table S5). We suspect that this enhancement is caused by active filtration in central forced-air heating systems. Noting that typical system runtimes are ~20% and assuming a MERV 11 filter efficiency of 50% and recirculation rates of ~4 h^−1^, a first-order estimate suggests that heating-associated filtration may increase particle loss-rate coefficients by 0.4 h^−1^ on a time-average basis (41, 42). During wintertime periods, we observe that particle loss-rate coefficients in the Cold climate zone are ~0.2 h^−1^ larger than loss-rate coefficients in other climate zones. With previously stated filtration efficiencies and recirculation rates, the Cold climate zone would require 10% more runtime than other climate zones on an absolute basis. While residences with central warm-air heating systems have the highest prevalence in the Cold climate zone (71%) as compared to Mixed/Hot-Dry (62%), Mixed-Humid (57%), Marine (54%), and Hot-Humid (46%) climate zones, differences in system runtimes among climate zones are not well characterized (41, 43). Enhanced evaporative losses associated with central heating may also contribute.

**Fig. 4.**
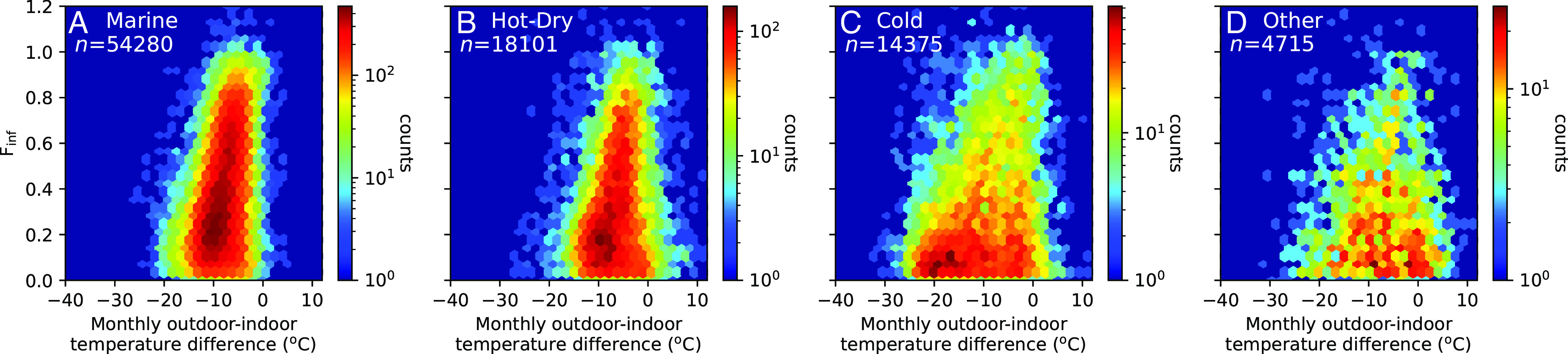
The PM_2.5_ infiltration factor is compared against the mean outdoor–indoor temperature difference at monthly time resolution. Monthly data points are binned into hexagons, with bin color corresponding to the total number of measurements. Panels correspond to data subsets specific to climate zones: (*A*) Marine, (*B*) Hot-Dry, (*C*) Cold, and (*D*) Other.

To explore the influence of weather on infiltration factors more directly, we compare monthly averages of outdoor–indoor temperatures against the monthly infiltration factor (Fig. 4 and *SI Appendix*, Fig. S3). When outdoor and indoor temperatures are comparable (outdoor minus indoor temperature in the range −10 to 0 °C), infiltration factors varied across the full range (0 to 1) with the highest density near $F_{inf}$ = 0.2. However, when the outdoor–indoor temperature difference was less than −10 °C (indicating a need for space heating) or higher than 0 °C (suggesting more likely use of air conditioning), the PM_2.5_ infiltration factors approximately halved in central tendency. These trends were observed across all climate zones, apart from cooling-associated losses being absent in the temperate Marine climate zone. This evidence indicates that active filtration associated with central air handling systems and/or behavioral changes in response to outdoor temperatures cause substantial changes in infiltration factors. One concern is that monthly outdoor–indoor temperature difference is an imperfect proxy variable for building operations that can affect the infiltration factor; the diel and seasonal temperature variations (*SI Appendix*, Figs. S4–S6) influencing window use, heating, and cooling are not fully captured using monthly average temperature data.

Building-specific year of construction data is not available in the National Structure Inventory. We used the median age of buildings within the same census tract as a proxy variable for house age with observations spanning decadal intervals: 1935–1945 (*n* = 675), 1945–1955 (*n* = 483), 1955–1965 (*n* = 757), 1965–1975 (*n* = 672), 1975–1985 (*n* = 639), 1985–1995 (*n* = 411), 1995–2005 (*n* = 251), and 2005–2015 (*n* = 22). We observed that residences located in census tracts with older homes were leakier than residences located in census tracts with newer homes (*SI Appendix*, Fig. S7). It has been reported that building envelope leakiness is related to building age (44). Infiltration factors decrease by 37% when comparing census tracts with median construction years of 1970 against census tracts with median construction years of 2010. Similarly, particle loss-rate coefficients decrease by 9% when comparing census tracts with median construction years of 1970 against census tracts with median construction years of 2010. Efforts to reduce the leakiness of building envelopes were initiated in the 1970s to improve energy efficiency and have continued both for new construction and in retrofits. The magnitude of these observed trends is much smaller than the substantial variation within each building age subgroup. The larger within-group variability may reflect uncertainty from the choice of building-age proxy variable or substantial variation within each building-age subgroup. Differences among building types were comparable between wood (*n* = 2,292, mean *F*_inf_ = 0.26) and masonry (*n* = 1,547, *F*_inf_ = 0.27) structured homes.

### Episodic Emission Analysis.

**Fig. 5.**
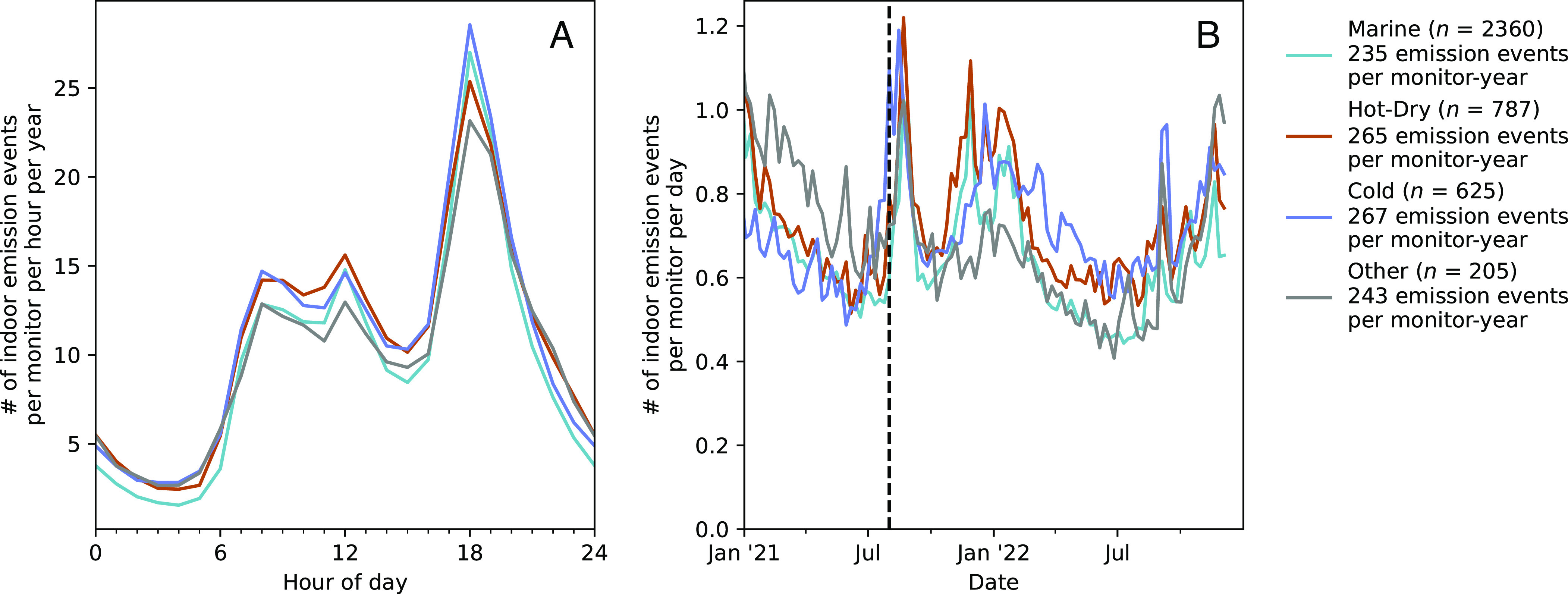
Summary of the temporal distribution of episodic indoor emission events. In panel (*A*), mean values for the number of indoor emission events occurring per monitor per year at a given hour of day are displayed. In panel (*B*), mean values for the number of indoor emission events occurring per monitor per day are displayed at weekly time resolution. The vertical hatched line on 1 August 2021 marks a period of wildfires in the western United States. In both panels, traces correspond to the mean value of each climate zone data subset. The integrated area of each panel is normalized and displayed in the legend alongside the number of residences in each climate zone.

We identified 1.3 million episodic indoor emission events where peak prominence surpassed 5 μg m^−3^ and peak width was less than 6 h. Accordingly, the mean and median sampled residences displayed 0.84 and 0.50 emission events per day, respectively, with 10th and 90th percentiles of 0.03 and 2.00 emission events per day, respectively. Indoor emission events were most frequent during common mealtimes (breakfast, lunch, and, most prominently, dinner) and least frequent during nights when residents are more likely to be sleeping (Fig. 5*A*). Moreover, the largest baseline departures in the number of indoor emission events were observed on holidays such as Thanksgiving (1.13 and 1.09 emission events per monitor-day in 2021 and 2022, respectively) and Christmas (1.00 emission events per monitor-day in 2021), especially for large emission events (>30 μg m^−3^) (*SI Appendix*, Fig. S8). Together, this evidence substantiates, on a larger scale, previous reports that human-related activities, especially cooking, are dominant sources of indoor episodic PM_2.5_ emissions (45). Indoor emission events were most frequent in winter and the least frequent in summer (Fig. 5*B*). This feature is explained, in part, by Western populations spending more time at home during the winter season (4) with more frequent cooking (46), as well as the occurrence of wintertime holiday gatherings such as Thanksgiving and Christmas. We also observed that more emission events occurred on weekends than weekdays (*SI Appendix*, Fig. S9). Accordingly, $C_{iie}$ and $C_{i}$ concentrations were 8% and 6% higher on weekends, respectively. Finally, more indoor emission events were observed during the 2021 wildfire period than expected based on the 2021 and 2022 seasonal trends (Fig. 5*B* and *SI Appendix*, Fig. S8), suggesting either a) occupants performed more activities at home during periods of high outdoor air pollution or b) wildfire plumes penetrated indoors with sufficient variability to register as an episodic peak. Misidentification of infiltrated wildfire plumes could bias our results.

We integrated the time-series concentration of each emission event (units of μg m^−3^ h) and multiplied the result by the particle loss-rate coefficient (median 1.2 h^−1^, mean 1.8 h^−1^ over full dataset). The result corresponds to the expected concentration increase if the entire episodic emissions event occurred instantaneously into a well-mixed interior volume. Distributions of instantaneous concentration enhancements so obtained can be found in *SI Appendix*, Fig. S9 with mean (21.2 μg m^−3^) and median (19.8 μg m^−3^) observed values. Instantaneous concentration enhancement distributions were greatest in the Cold climate zone and were indistinguishable among Marine, Hot-Dry, and Other zones.

After restricting the dataset to single-family residences with area less than 560 m^2^ (3,423 of 3,977 total residences), we estimated residential volumes from these areas and an assumed room height of 2.4 m. Using residential volumes, we estimated episodic emissions in units of mass emitted for each event. The resulting arithmetic mean and median were 22.6 and 8.3 mg, respectively (*SI Appendix*, Fig. S10). These emission analyses are limited in that they assume a single instantaneously well-mixed zone and do not consider spatial heterogeneity, mixing times, or decoupled zones. Event-driven concentration enhancements were inversely related to home floor area (*SI Appendix*, Fig. S11*A*), as expected given the larger dilution volume. The calculated total emission mass increased with home area (*SI Appendix*, Fig. S11*B*). Chan et al. (47) conducted similar time-series analysis on concentration data from 18 California residences. The mean and median peak emissions (30 and 12 mg, respectively) and decay rates (1.3 and 2.0 h^−1^, respectively) in Chan et al. (47) and a related study by Sun and Wallace (39) are harmonious with our findings from the much larger dataset, with about 3 orders of magnitude more residence-days of monitoring.

### Health Guideline Comparisons.

The World Health Organization (WHO) annual air-quality guideline recommends that PM_2.5_ be maintained at annual mean concentrations no higher than 5.0 μg m^−3^ and the WHO daily air-quality guideline recommends that the 99th percentile of daily mean concentrations be no higher than 15.0 μg m^−3^. These guidelines were developed from outdoor epidemiological studies and are intended to apply to both outdoor and indoor settings. Although these guidelines are not legally binding and indoor measurements may be influenced by sensor calibration reliability, a comparison of the indoor measurements with the guidelines is informative about potential health risks associated with indoor PM exposure. Among the almost 4,000 residences monitored, exceedances of the annual PM_2.5_ guidelines were observed for 484 residences (12% of total). Exceedances of the daily PM_2.5_ guidelines were observed at 1,480 residences (37% of total).

We note that the PurpleAir sensor network does not reflect a representative sample of the US building stock. Previous research has demonstrated that PurpleAir monitors are more likely to be found in homes with higher valuations than those of neighbors. Environmental justice communities with greater health and pollutant burdens are likely to be underrepresented within the dataset (33, 48, 49). Care is needed in generalizing these findings to the US population.

### Implications.

This study found that approximately half of residential PM_2.5_ concentrations originate from indoor sources, consistent with historical studies conducted on far smaller datasets. By studying thousands of residences in detail, this study observed substantial heterogeneity in source contribution with indoor-generated fractions of residential PM_2.5_ varying between roughly one-sixth and three-fourths of total PM_2.5_ (10th–90th percentiles) among residences. Identified features influencing this heterogeneity include occupant actions in building operation, cooking intensity, and building age and structure. These factors cumulatively indicate that both occupants and the building industry have substantial opportunities to reduce residential PM_2.5_ exposures. For example, infiltration factors decreased by half during periods of high outdoor air pollution and during wintertime, likely in association with enhanced filtration and a tighter building envelope as doors and windows remain closed. Even as concentrations of both persistent and episodic indoor-generated PM_2.5_ were observed to increase in response to the tighter building envelope, occupant actions reduced potential exposures to both outdoor PM_2.5_ and total PM_2.5_ with likely net benefit.

We also identified the frequency, magnitude, and timing of indoor emission events by season. Emission events were more frequent during mealtimes, food-centered holidays, weekends, and wintertime, cumulatively substantiating that cooking is a dominant source of episodic PM_2.5_. Enhanced source control measures such as the use of range hoods, filtration, or ventilation during cooking yield opportunities to reduce occupant exposures from the episodic emission events that contribute slightly more than half of indoor-generated PM_2.5_. While indoor sources are commonly assumed to be dominated by episodic events like cooking, this study also identified that persistent indoor sources are a major source, contributing nearly half of indoor-generated PM_2.5_. Persistent indoor sources may include contributions from chemical reactions or phase-change phenomena or continuous particle resuspension events that do not form discrete peaks or enhancements. For example, higher indoor concentrations of gaseous SVOC relative to the outdoors can lead to condensation of indoor SVOC on outdoor particles after outdoor-to-indoor particle transport (50).

Finally, we note that detection efficiencies for optical particle counters, including the Plantower particle sensor used by PurpleAir monitors, rapidly decline for particles smaller than about 300 nm. Cooking emission events can release large amounts of ultrafine particle mass (10), and low-cost sensors can miss ultrafine emission events entirely (51, 52). Even as we highlight the importance of indoor sources, indoor episodic concentrations, and therefore the total importance of indoor sources, may be underestimated in this work.

## Materials and Methods

### Data Selection.

PurpleAir monitor metadata and PM_2.5_ concentration time series were obtained from the PurpleAir sensor network. Monitor metadata were supplemented with building information from the National Structure Inventory (US Army Corps of Engineers, www.hec.usace.army.mil/confluence/nsi) and climate zone delineations used by the Building America program (US Department of Energy, https://www.energy.gov/eere/buildings/climate-zones). PurpleAir monitors provide optical measurements of PM_2.5_, temperature, and humidity, as well as GPS location coordinates and indoor–outdoor placement type. The final dataset consisted of 281 million paired indoor–outdoor PM_2.5_ measurements at 3,977 residences spanning 10,688 monitor-years of data with 10-min time resolution. These data span 39 US states, with most monitors located in west coast US states (3,345 of 3,977 residential monitors in CA, WA, or OR). Sampled locations include representation of all 7 Building America climate zones with most monitors located in the Marine, Hot-Dry, or Cold climate zones (3,772 of 3,977 residential monitors). The final dataset was constructed by 1) selecting all US indoor PurpleAir monitors from 2021 and 2022, 2) linking indoor monitors to the closest structure in the National Structure Inventory and restricting the dataset specifically to residences, 3) linking indoor residential monitors to the average of all outdoor monitors within 5 km, 4) removing monitors and/or data points that do not pass quality assurance and quality control tests, and 5) applying the “ALT” calibration model to all PM_2.5_ data. *SI Appendix* provides a detailed description for each of these steps.

### Time-series Analysis.

We estimated indoor episodic PM_2.5_ concentrations using a semiquantitative algorithm designed to identify episodic emission events related to indoor activities such as cooking, cleaning, or resuspension (*SI Appendix*, Fig. S1). More details are available in *SI Appendix*. We also calculated a first-order particle loss-rate coefficient for all particle emission events with well-behaved decay curves (*SI Appendix*, Fig. S12). The loss-rate coefficient ( $\lambda_{tot}$ ) combines contributions from the air-change rate ( a ), the particle deposition rate ( $k_{dep}$ ), and, if present, active particle filtration ( $k_{filt}$ ). More details are available in *SI Appendix*.

### Random Component Superposition Analysis.

The random component superposition (RCS) method regresses time-averaged indoor PM_2.5_ concentrations against time-averaged outdoor PM_2.5_ concentrations. Resulting fitted parameters are interpreted as the infiltration factor (slope = $F_{inf})$ and indoor concentrations attributable to indoor sources (intercept = $C_{ii}$ ). We applied the RCS model using daily averaged concentration data over the full dataset, yielding singular values for each building, as well as over subsets by month, yielding results at monthly time resolution for each building. We also introduce a modified form of RCS analysis to differentiate indoor concentrations attributable to episodic emissions from indoor persistent sources ( $C_{iip})$ and to correct potential biases associated with ordinary least squares regression. In modified RCS, we generated excised indoor concentration data where peaks are removed using a peak-finding algorithm. We then regress the excised indoor concentration data against outdoor concentration data using orthogonal distance regression, yielding fitted parameters interpreted as the infiltration factor (slope) and indoor concentrations attributable to indoor persistent sources (intercept = $C_{iip}$ ) (*SI Appendix*, Figs. S13–S15). Unless otherwise specified, all RCS results are reported using the modified RCS model which was applied over the full dataset as well as subsets by month. More details are available in *SI Appendix*.

## Supplementary Material

Appendix 01 (PDF)

## Data Availability

Study data can be acquired from publicly available sources at PurpleAir (https://api.purpleair.com) (53) and the National Structure Inventory (https://www.hec.usace.army.mil/confluence/nsi) (54). PurpleAir was founded on an open science model with data made publicly available; PurpleAir data may be shared with third parties subject to permission from PurpleAir.
