# Supplementary material for: Assessing residential PM2.5 concentrations and infiltration factors with high spatiotemporal resolution using crowdsourced sensors
Source: Proc Natl Acad Sci U S A. 2023 Dec 4;120(50):e2308832120. doi: 10.1073/pnas.2308832120 (PMC10723120; doi:10.1073/pnas.2308832120)
Supplement: Supplementary file 1 — Appendix 01 (PDF) [file pnas.2308832120.sapp.pdf]

PNAS

Supporting Information for

Assessing residential $PM_{2.5}$ concentrations and infiltration factors with high spatiotemporal resolution using crowdsourced sensors.

David M. Lunderberg,[1,2] Yutong Liang,[1,3] Brett C. Singer,[4] Joshua S. Apte,[5,6] William W Nazaroff,[5] Allen H. Goldstein[1,5]

[1]Department of Environmental Science, Policy, and Management, University of California, Berkeley, CA, USA.
[2]Department of Chemistry, University of California, Berkeley, CA, USA.
[3]School of Chemical and Biomolecular Engineering, Georgia Institute of Technology, Atlanta, GA, USA.
[4]Indoor Environment Group, Lawrence Berkeley National Laboratory, Berkeley, California, USA.
[5]Department of Civil and Environmental Engineering, University of California, Berkeley, CA, USA.
[6]School of Public Health, University of California, Berkeley, CA, USA.

*David M. Lunderberg
Email: david_lunderberg@berkeley.edu

This PDF file includes:

Supporting text
Figures S1 to S15
Tables S1 to S7
SI References

EXTENDED MATERIALS AND METHODS

Notation.
C_{o} = outdoor $PM_{2.5}$ concentration
C_{i} = indoor $PM_{2.5}$ concentration
C_{io}= indoor $PM_{2.5}$ concentration of outdoor origin
C_{ii} = indoor $PM_{2.5}$ concentration of indoor origin
C_{iie}= indoor $PM_{2.5}$ concentration of indoor episodic origin
C_{iip}= indoor $PM_{2.5}$ concentration of indoor persistent origin
λ_{tot} = first-order loss-rate coefficient for episodic particle emissions
$\bar{\lambda}_{\mathrm{tot}}$ = mean of loss rate coefficients for episodic particle emissions at one residence
F_{inf} = infiltration factor
RCS = random component superposition
NSI = National Structure Inventory
$PM_{2.5}$ = mass concentration of particles smaller than 2.5 micrometers in diameter
Potential exposure = the time-integrated concentration of a hypothetical occupant who is always inside the studied residence.

Data acquisition. Our process began by selecting all indoor PurpleAir monitors with at least one outdoor monitor within 5 km, yielding 5617 indoor monitors. We gathered building metadata for the closest structure from the National Structure Inventory (US Army Corps of Engineers, www.hec.usace.army.mil/confluence/nsi) using the GPS coordinates associated with each indoor monitor. We removed monitors from the analysis set if the closest structure in the National Structure Inventory was more than 50 m away, yielding 4884 indoor monitors. We further restricted the analysis to buildings specifically identified as residences, reducing the dataset to 4075 indoor monitors. Finally, we removed 98 indoor monitors that contained less than one month of paired indoor-outdoor data or appeared to be outdoor monitors mislabeled as indoors. Mislabeled monitors were identified by regressing the $PM_{2.5}$ concentrations reported by an identified indoor monitor against those of the nearby outdoor monitors. Monitors coded as “indoor” whose regressions returned slopes greater than 0.9 and coefficients of determination (R^2) greater than 0.85 relative to the nearby outdoor monitors were redefined as being outdoors and excluded from the analysis. The final dataset included 3977 indoor monitors with at least 1-month of paired indoor and outdoor concentrations at 10-min time resolution, with the indoor monitor specifically monitoring a residence in the contiguous United States. Outdoor $PM_{2.5}$ concentrations were defined as the arithmetic mean of all outdoor monitors within 5 km of each indoor monitor for each time point.

We followed the QA/QC procedures reported by O’Dell et al. (1) for the Plantower sensors used by PurpleAir monitors. First, we removed indoor datapoints with $PM_{2.5}$ concentration outside the range 0–500 μg m^{-3} using the default CF1 calibration provided by Plantower, which corresponded to 0.15% of the original indoor dataset. Second, we removed data points where reported temperatures and humidities were outside of standard operating ranges (14–140 °F and 0–99% relative humidity), corresponding to 0.02% of the original indoor dataset. Third, we removed datapoints for monitors with two sensors in the same device when the two sensors differed by > 10 μg m^{-3} (absolute concentrations < 100 μg m^{-3}) or > 10% (absolute concentrations ≥ 100 μg m^{-3}), which corresponded to 0.14% of the original indoor dataset. This step was omitted for monitors with only one sensor in the device. For monitors with more than one sensor, values are reported as the average of both sensors. Finally, we omitted

indoor datapoints for which there was no concurrent outdoor measurement. In total, 1.2% of the original indoor $PM_{2.5}$ dataset was excluded from analysis.

The Plantower particle sensor reports number concentrations for six size ranges based on minimum particle size (i.e., >0.3 µm, >0.5 µm, >1 µm, >2.5 µm, >5 µm and >10 µm) and includes proprietary algorithms to calculate and report mass concentrations of PM_1, $PM_{2.5}$ and PM_{10}. Correction factors to these proprietary algorithms have been developed by comparing outdoor PurpleAir monitors with outdoor EPA monitoring stations, some of which account for changes in humidity. Open algorithms have also been developed that calculate $PM_{2.5}$ from particle number concentrations and have similarly been validated against outdoor EPA monitoring stations. In this work, we quantified data using the "ALT" algorithm (2, 3) because it shows the best performance for low concentrations. We note that the ALT calibration used in this work was validated against outdoor $PM_{2.5}$ monitoring stations; it has not been validated in indoor settings. A related concern is that the variable particle densities of outdoor aerosol (higher density), cooking aerosol (lower), and resuspended aerosol (higher) are not considered in this work or in calibration, potentially biasing results. We also highlight that the exact locations of PurpleAir monitors within residences are not known and may not be fully representative of conditions within the home, such as a monitor placed in a closed bedroom during kitchen cooking. Apparent loss from dilution may cause loss rates to be overestimated if monitors are placed near a source. Considering potential exposures, the concentrations measured in residences will tend to underestimate the concentration directly at the breathing zone due to proximity and personal cloud effects (4). Finally, the performance of Plantower sensors is known to degrade over time (5). This feature is difficult to evaluate for models of PurpleAir monitors with only one Plantower sensor.

While temperatures and humidities reported by the PurpleAir monitors correlate well with reference monitors, such parameters are biased slighter warmer and drier than reference monitors (6), impacting conditions reported in Figures S4-S6.

We collected other building metadata from the National Structure Inventory, including the structure type (commercial, residential single-family, residential multi-family), structure floor area (square footage), structure replacement value (in dollars), and the median year built of structures within the same census block, among other parameters.

We also identified climate zones as defined by US DOE's Building America Program (US Department of Energy, https://www.energy.gov/eere/buildings/climate-zones) using the geographic coordinates of each monitor. The Building America Project defines seven climate zones based on temperature and precipitation: "Very Cold," "Cold," "Marine," "Mixed-Dry," "Mixed-Humid," "Hot-Dry," and "Hot-Humid."

We collected population density data for 40,954 US zip codes (https://www.fourfront.us/data/datasets/us-population-density/). To connect these population density estimates with residential monitors, we identified the zip code of each residential monitor using the monitor's GPS coordinates and the Nominatim API for OpenStreetMap (nominatim.openstreetmap.org). When Nominatim was unable to return a US zipcode or returned an invalid zip code, we manually identified the monitor's zipcode using Google Maps.

PurpleAir monitors report data times in UTC. To identify local time, we linked GPS coordinates of each monitor to the closet US city with known time zone. The time zones of US cities were acquired from the GeoNames database (http://download.geonames.org/export/dump/cities15000.zip).

Time series analysis. A semiquantitative algorithm was developed to identify the start bound of an event and the stop bound when the event's impact has concluded. The algorithm was visually assessed to ensure satisfactory performance (Figure S1). The event start bound was defined as the transition point from negative or fluctuating first derivative to predominantly positive first derivative. The algorithm begins by scanning point-by-point leftward (back in time) from the event concentration peak towards the event start. Each step corresponds to 10 min. During iteration, two counters mark the cumulative number of datapoints with negative first derivative, denoted *a*, and the number of consecutive datapoints with negative first derivative, denoted *b*. The second counter, *b*, increases by one with an instance of negative derivative and resets to zero with an instance of positive first derivative. For well-behaved events, the first counter, *a*, remains near zero until the event start has been reached. Iteration continues until the first counter, *a*, reaches a value of seven. To avoid going beyond the event start bound, the event start bound is defined as *b* datapoints to the right of the iteration stop datapoint. The event stop bound is similarly defined as the transition point from predominantly negative first derivative to positive or fluctuating first derivative and implements the same algorithm. The algorithm begins by iterating rightward (forward in time) from the event concentration peak. Two counters mark the cumulative number of datapoints with positive first derivative, denoted *c*, and the number of consecutive datapoints with positive first derivative, denoted *d*. Iteration continues until the first counter, *c*, reaches a value of seven. The event stop bound is then defined as *d* datapoints to the left of the iteration stop datapoint.

Indoor baseline concentrations with events removed were estimated by interpolating between the event start and stop bounds. Additionally, for time periods where indoor baseline concentrations exceeded outdoor concentrations, the outdoor concentration was defined as the indoor baseline. We manually examined hundreds of weeks of data to ensure satisfactory performance. Event start and stop bounds were ambiguous for a notable minority of peaks, especially for small peaks during times with large outdoor concentration.

A prior study (7) defined concentration thresholds of greater than 30 μg m^{-3} and width thresholds of less than 4 h to be of unambiguous indoor origin. Indoor emission peaks with widths larger than 4 h are rarely present and are often indicative of an extended outdoor emission source such as a wildfire. However, many indoor source events, such as those related to resuspension or light cooking are be captured when using the 30 μg m^{-3} concentration threshold. We conducted sensitivity testing to determine whether the 30 μg m^{-3} threshold may underestimate the total contributions of indoor episodic emissions to indoor $PM_{2.5}$. Averaging over the total dataset, the 30 μg m^{-3} threshold captures 90.3% of the total integrated peak area when compared to a threshold of 5 μg m^{-3}. One concern may be potential overcounting where episodic outdoor emission events lead to smaller episodic indoor concentration enhancements that are misidentified as being of indoor origin. This concern is ameliorated by the time-lag for outdoor concentrations to penetrate indoors, thereby dampening peak height and making misidentification less likely. Furthermore, visual

analysis suggests that while outdoor emission events leading to misattributed indoor concentration enhancements do occur, they are uncommon and the potential bias smaller in magnitude than would occur with a 30 μg m^{-3} peak threshold for most time periods. The potential bias may be important during wildfire events with high outdoor $PM_{2.5}$ concentrations.

To assess particle loss rates, we calculated the first-order loss-rate coefficient (λ_{tot}) of emission events with well-behaved decay curves. Specifically, we selected all peaks with a prominence of at least 5 μg m^{-3} and a peak duration between 20 min and 6 h.

The start of the decay curve fit region was defined as the event concentration maximum. The range of the decay curve fit region was defined as three times the peak width, where the peak width is the full horizontal peak width at half-prominence peak height. For each selected peak, we calculated the exponential decay using measurement points from the peak maximum to the measurement point that was 3× the peak width after the peak maximum (Figure S12). Peak fits with $R^2 > 0.8$ were defined as well-behaved while peaks with $R^2 < 0.8$, signifying poor fit quality, were rejected from further analysis as poorly behaved. The loss-rate coefficient (λ_{tot}) has contributions from indoor-to-outdoor transport, deposition, and, potentially, filtration. A mass balance equation is shown in Equation S1 for the decay of an emission event without source terms, where C_{ind} is the indoor concentration, a is the air-change rate, k_{dep} is the particle deposition rate, k_{filt} is the particle filtration rate, and $\delta(t)$ is a modifying function that is 0 when filtration is inactive and 1 when filtration is active. This simplified mass balance equation assumes that source terms (persistent indoor emissions and outdoor-to-indoor transport) are small after major indoor emission events and that the episodic emission source has completely ended.

$$\frac{dC_{\text{ind}}}{dt} = -\lambda_{tot} \times C_{\text{ind}}(t) \quad \text{(S1)}$$

$$\lambda_{tot} = a + k_{\text{dep}} + \delta(t) \times k_{\text{filt}} \quad \text{(S2)}$$

Peaks were integrated yielding peak area in units of μg m^{-3} h. We multiplied the integral area by either (a) their specific loss-rate coefficient for well-behaved peaks or (b) the average loss-rate coefficient over the monitor's total dataset for poorly behaved peaks, yielding the instantaneous concentration enhancement in units of μg m^{-3}. This value corresponds to the expected concentration increase if event emissions occurred instantaneously in a well-mixed interior volume. We multiplied this value by building volume to obtain episodic emission strengths in units of mg. Residence volumes were estimated from structure floor area (square footage) as reported by the National Structure Inventory and an assumed floor height of 2.4 m. Because the National Structure Inventory reports total building area rather than residential area, emission mass analysis was restricted to single-family residences (3458 of 3977 residences) where it is more justifiable to apply the well-mixed assumption over the total residential volume.

Random component superposition analysis. Random component superposition (RCS) analysis (8) was used to estimate infiltration factors and $PM_{2.5}$ attributable to indoor sources. Briefly, the method assumes that indoor $PM_{2.5}$ is linearly composed of indoor sources and outdoor $PM_{2.5}$, with the latter scaled by an attenuation factor related to penetration and deposition rates. It is further assumed that the indoor and outdoor sources are independent.

Then, fitted parameters from the regression of indoor $PM_{2.5}$ concentrations against outdoor $PM_{2.5}$ concentrations are interpreted as the infiltration factor (slope = F_{inf}) and the concentration attributable to indoor sources (intercept = C_{ii}). RCS analysis was performed for each indoor monitor separately, using 1-day time averaged concentrations on datasets with at least 27 days of data.

We also conducted a modified RCS analysis by applying the same procedure on an excised indoor concentration time series where episodic peaks were removed. Fitted parameters from the regression of excised indoor $PM_{2.5}$ against outdoor $PM_{2.5}$ can be interpreted as the infiltration factor (slope = F_{inf}) and the indoor source concentration attributable to persistent indoor emissions (intercept = C_{iip}). A visual demonstration of both methods is provided in Figure S13.

We conducted two tests to ensure internal validity of the modified RCS model. These tests are not an external validation of the method. First, the difference between C_{ii} as returned by RCS and C_{iip} as returned by modified RCS should be C_{iie}. A scatterplot of $C_{\text{ii}} - C_{\text{iip}}$ and C_{iie} demonstrates nearly 1-to-1 comparability (Figure S14). Second, we compare infiltration factors returned by RCS and modified RCS. A scatterplot of F_{inf} (RCS) and F_{inf} (modified RCS) similarly demonstrates nearly 1-to-1 comparability (Figure S15) when only considering physical values between 0 and 1 (coefficient of determination = 0.86, linear fit slope = 0.83). We note that the performance of modified RCS is seemingly improved, with all values of F_{inf} bounded at appropriate physical limits (between 0.003 and 1.002), whereas the original RCS method yields outliers with non-physical values, spanning a range from -2.2 to 7.0. Additionally, the median coefficient of determination (R^2) during analysis improved from 0.27 (RCS) to 0.58 (modified RCS) and the median root mean square error improved from 2.3 $\mu g\ m^{-3}$ (RCS) to 1.1 $\mu g\ m^{-3}$ (modified RCS). A final consideration is the potential for systemic errors in relation to the regression method. When uncertainty is present in both the independent and dependent variables, as is the case in RCS analysis, ordinary least squares regression can underestimate the slope and overestimate the intercept (9). We therefore apply orthogonal distance regression (Deming regression with $\delta = 1$) in modified RCS analysis to avoid this potential bias. A comparison of orthogonal distance regression against ordinary least squares regression finds that the population mean of F_{inf} increased by 6% (0.26 to 0.28, $R^2 = 0.99$) and population mean of C_{iip} decreased by 14% (0.63 to 0.55 $\mu g\ m^{-3}$, $R^2 = 0.97$) across all indoor-outdoor pairs. Values of F_{inf} remain bounded by the appropriate physical limits. Because analysis by modified RCS with orthogonal distance regression appears more robust than traditional RCS, we (a) report F_{inf} values from modified RCS with orthogonal distance regression and (b) report C_{ii} as the sum of C_{iie} and C_{iip} throughout the work unless otherwise specified.

As a time-integrated mechanistic model, RCS analysis makes a simplifying assumption that indoor source contributions and the infiltration factor are independent. The model does not consider how concentration-dependent variations in particle composition and phase-change phenomena may systematically bias results. For example, ammonium nitrate and other aerosols can partially evaporate upon transport to the indoors (10, 11, 12). If these processes occur more readily during periods of high outdoor concentrations than low outdoor concentrations, the net effect would be to flatten the slope (decrease F_{inf}) and increase the intercept (increase C_{ii} or C_{iip}) in either RCS or modified RCS analysis. Ammonium nitrate

aerosols are a substantial contributor to outdoor $PM_{2.5}$ concentrations, especially in urban areas during wintertime when outdoor $PM_{2.5}$ concentrations are high (13, 14). We observe the highest C_{iip} concentrations in wintertime. While it is plausible that elevated C_{iip} concentrations are related to wintertime tightening of the building envelope (e.g., from less window use), alternative hypotheses such as concentration-dependent nitrate evaporation may also contribute. Phase-change phenomena influencing the indoor-outdoor fine particle relationship are potentially important for nitrates, semivolatile organics, and water. Similar effects related to concentration-dependent calibration biases may also influence RCS model results.

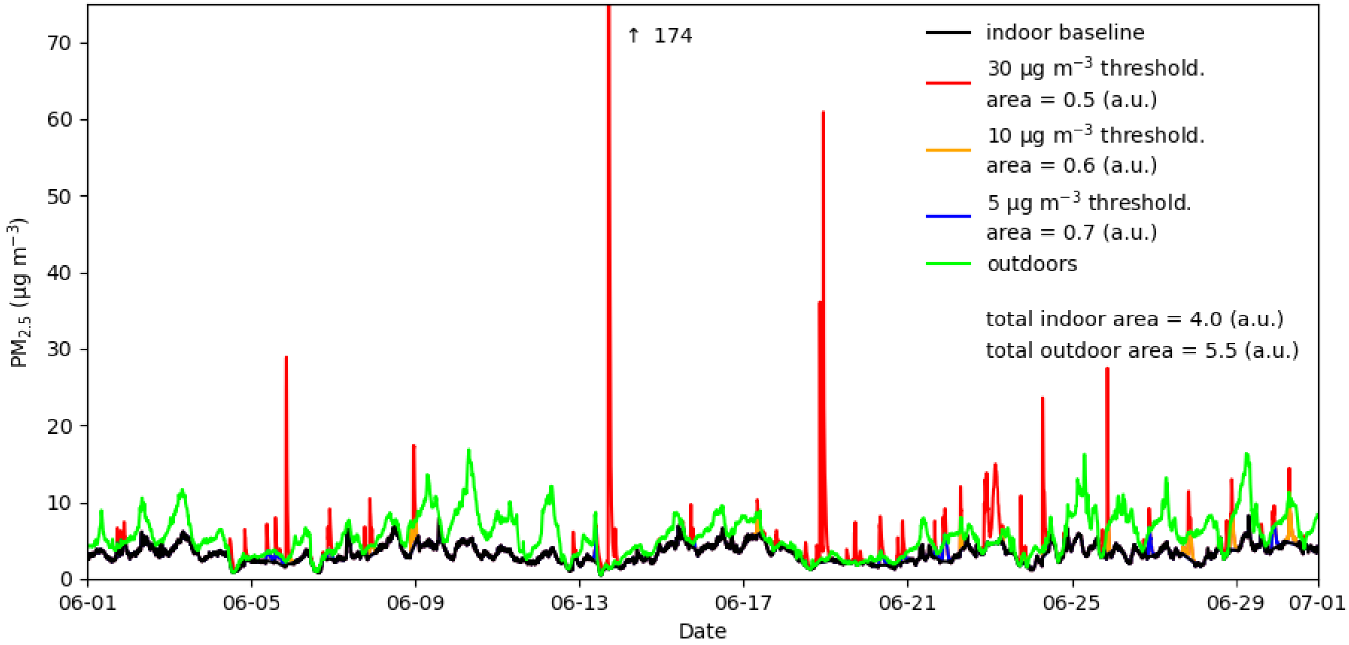

Figure S1. A representative concentration time series is displayed. Indoor concentrations captured by the semiquantitative peak-finding algorithm using a 30 µg m^{-3} peak prominence threshold (or when indoor concentrations are higher than outdoors) are shown in red. Additional peaks captured by 10 and 5 µg m^{-3} thresholds are shown in orange and blue, respectively. The indoor baseline is displayed in black and outdoor concentrations are displayed in green. Integrated areas are reported in arbitrary units (a.u.).

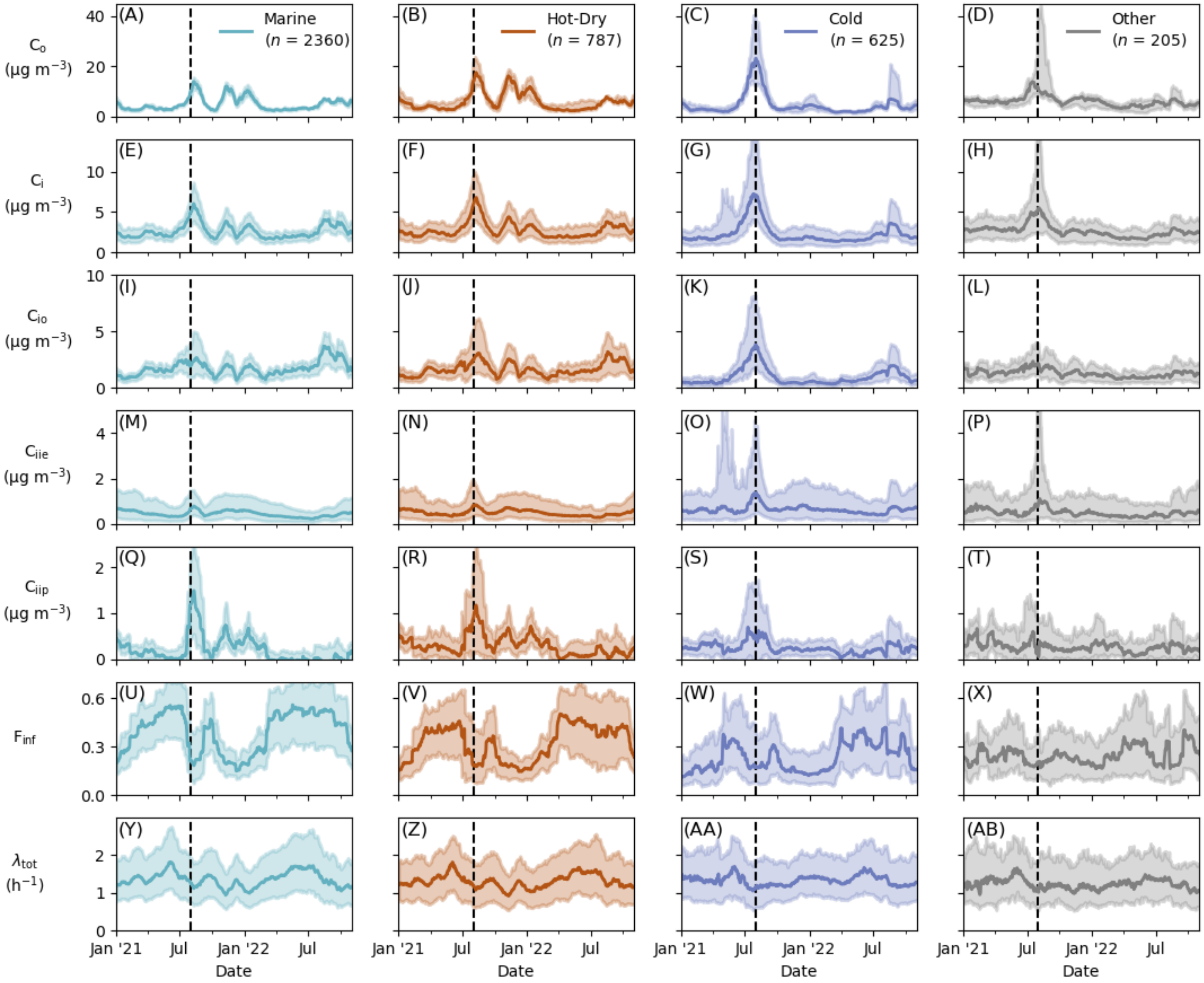

Figure S2. Time series of outdoor $PM_{2.5}$ (µg m^{-3}), indoor $PM_{2.5}$ (µg m^{-3}), indoor $PM_{2.5}$ of indoor episodic origin (µg m^{-3}), indoor $PM_{2.5}$ of indoor persistent origin (µg m^{-3}), infiltration factors (unitless), and residence-specific mean loss-rate coefficients (h^{-1}) are displayed in rows. Data specific to a climate zone are displayed in columns. Datapoints forming the solid line correspond to the median value of all indoor-outdoor pairs after re-sampling individual monitors to a rolling 30-day window and then grouping by climate zone. Shaded regions indicate the interquartile ranges. The vertical hatched line on 1 Aug. 2021 marks a period of extensive wildfires in the western United States.

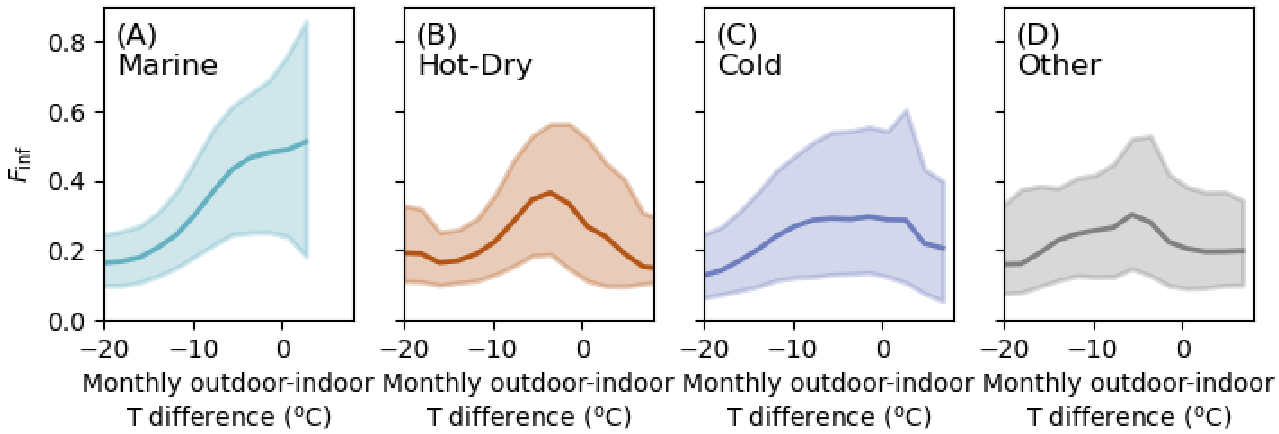

Figure S3. The median infiltration factor F_{inf} is displayed as a function of monthly outdoor minus indoor temperature (°C) for each climate zone in panels A–D. Shaded regions indicate the interquartile ranges. Temperature regions with fewer than 50 aggregate observations are not displayed.

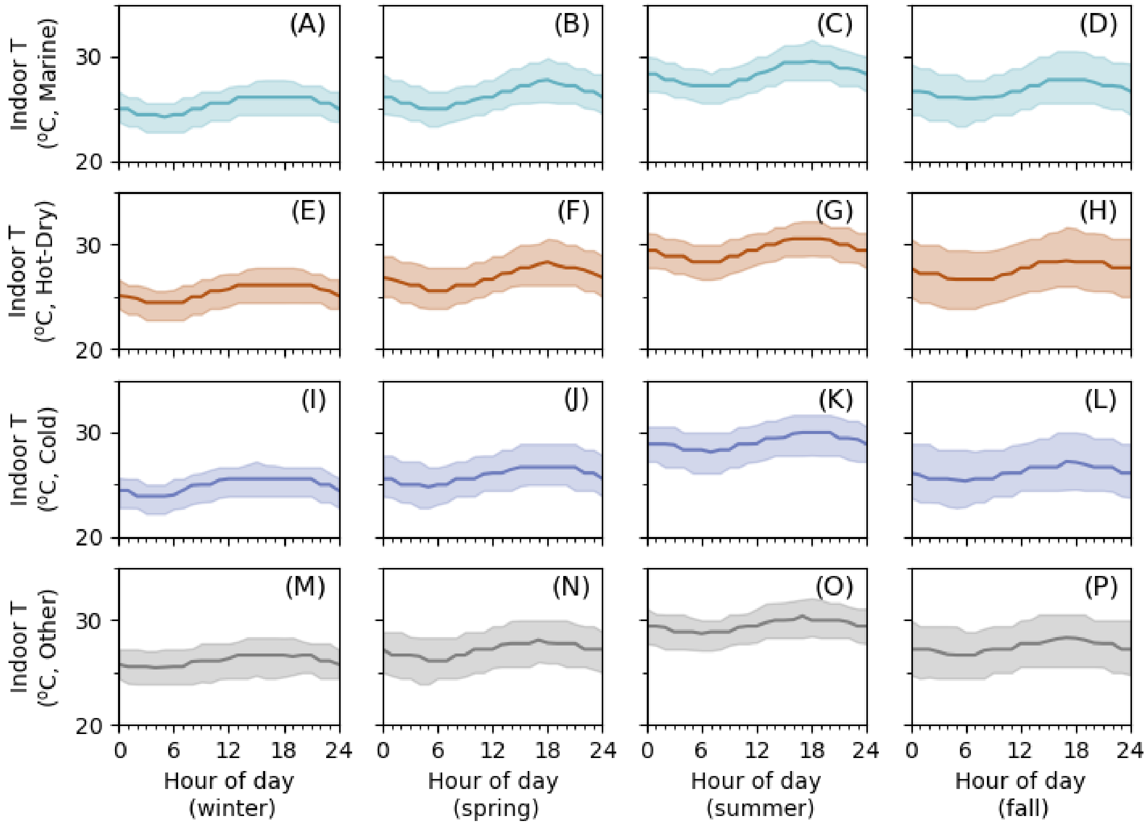

Figure S4. Diel plots of indoor temperature (°C) are displayed by season (columns) and climate zones (rows). Plotted values correspond to the median value of the median observed temperature over all selected monitors. Shaded regions correspond to the median value of the 25th and 75th percentile observed temperatures over all selected monitors.

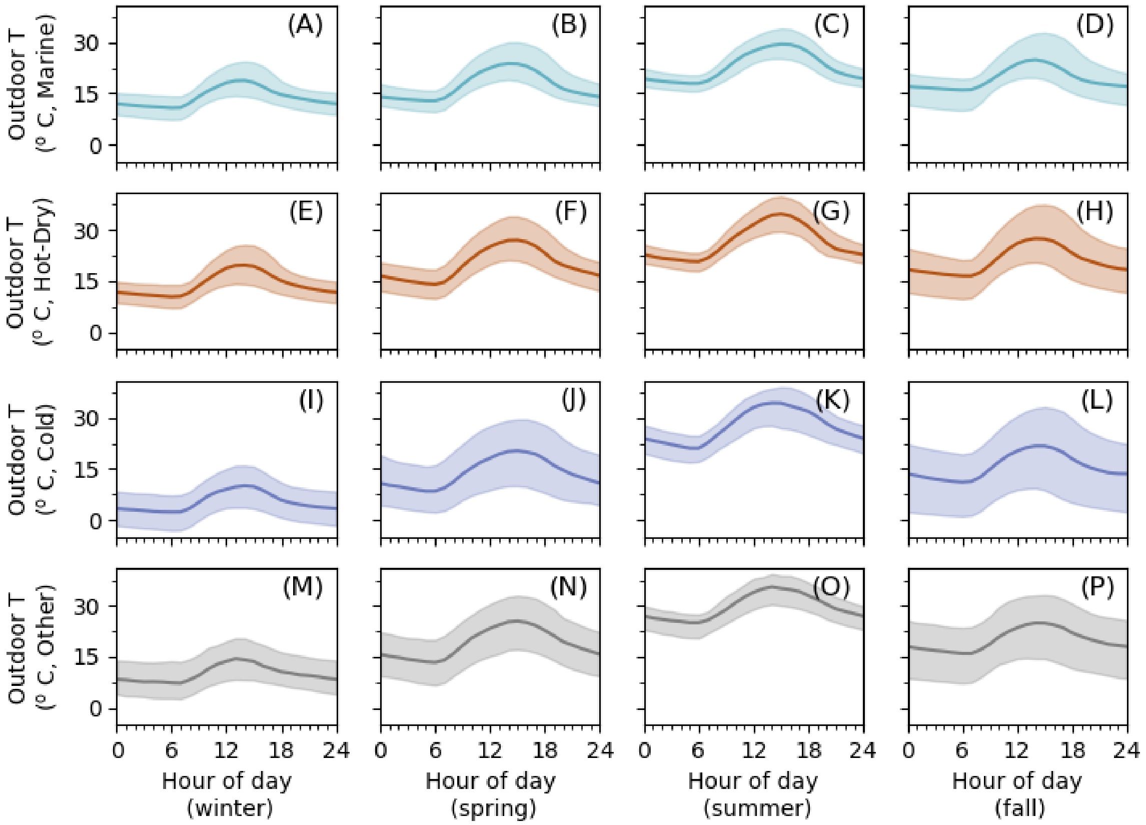

Figure S5. Diel plots of outdoor temperature (°C) are displayed by season (columns) and climate zones (rows). Plotted values correspond to the median value of the median observed temperature over all selected monitors. Shaded regions correspond to the median value of the 25th and 75th percentile observed temperatures over all selected monitors.

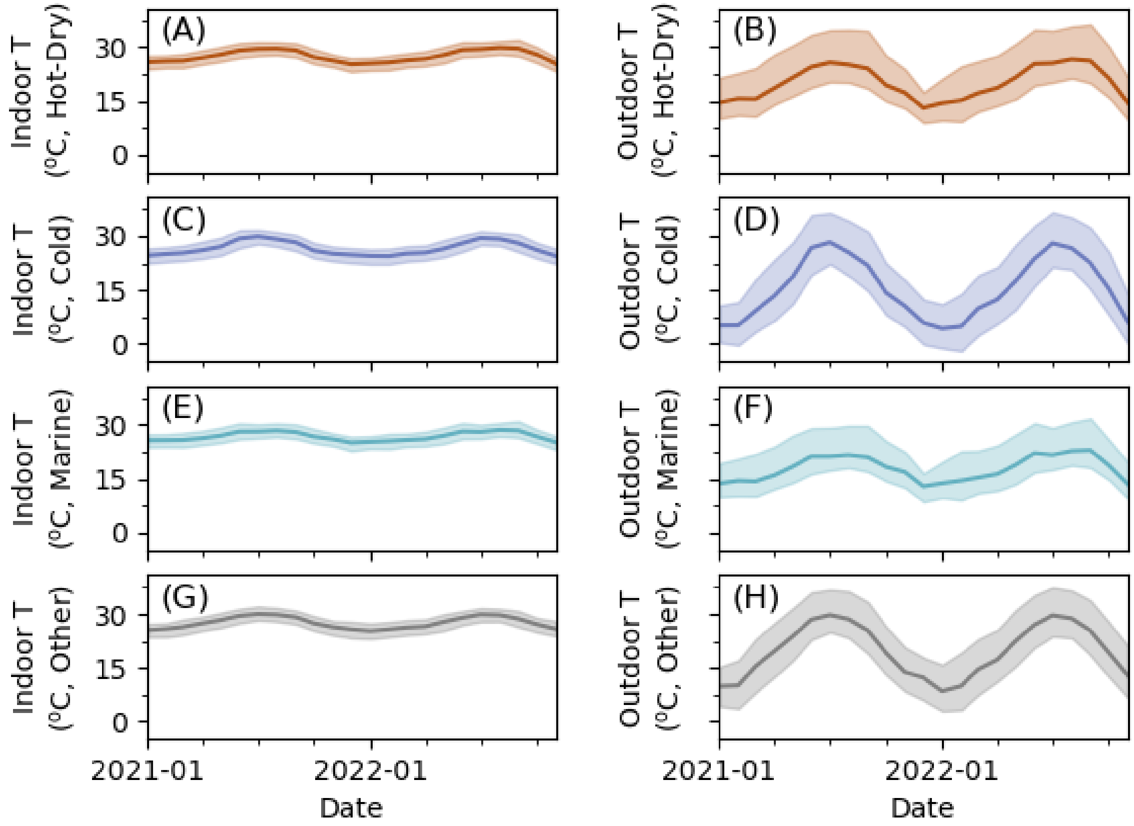

Figure S6. Time series of indoor temperatures (left column, °C) and outdoor temperatures (right column, °C) are displayed for each climate zone (rows) at monthly time resolution. Plotted values correspond to the median value of the median observed temperature over all selected monitors. Shaded regions correspond to the median value of the 25th and 75th percentile observed temperatures over all selected monitors.

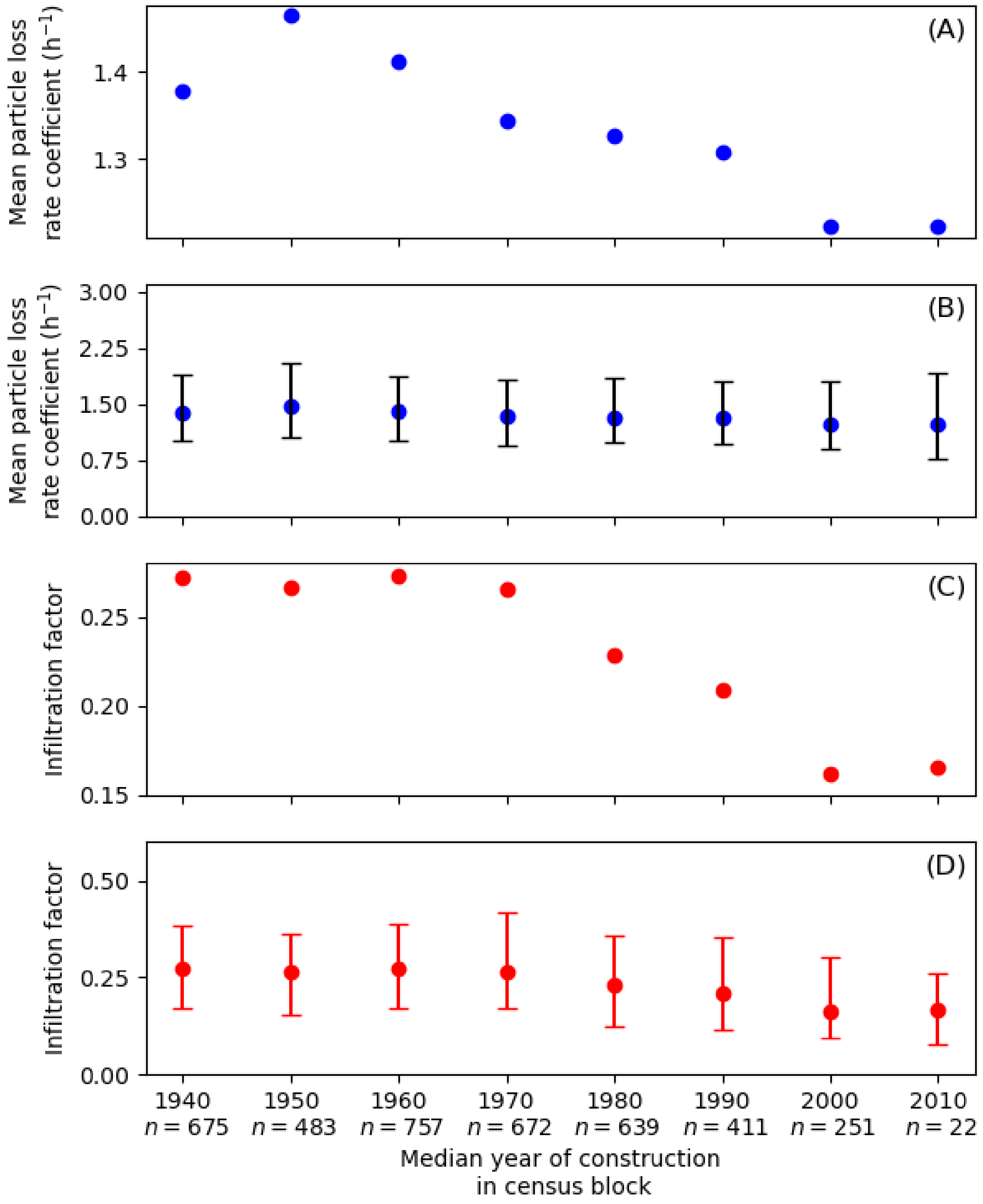

Figure S7. Summary statistics for mean particle loss-rate coefficients and calculated infiltration factors for residences. Individual residences were linked to the median year of construction of all structures within a residence's census block (not limited to those with PurpleAir data) and then grouped by decade. Panels A (particle loss rates) and C (infiltration factors) present the median values of each decadal grouping with the *y*-axis spanning the minimum and maximum values. Panels B (particle loss rates) and D (infiltration factors) present the same data with the addition of the interquartile range and with the *y*-axis beginning at *y*=0.

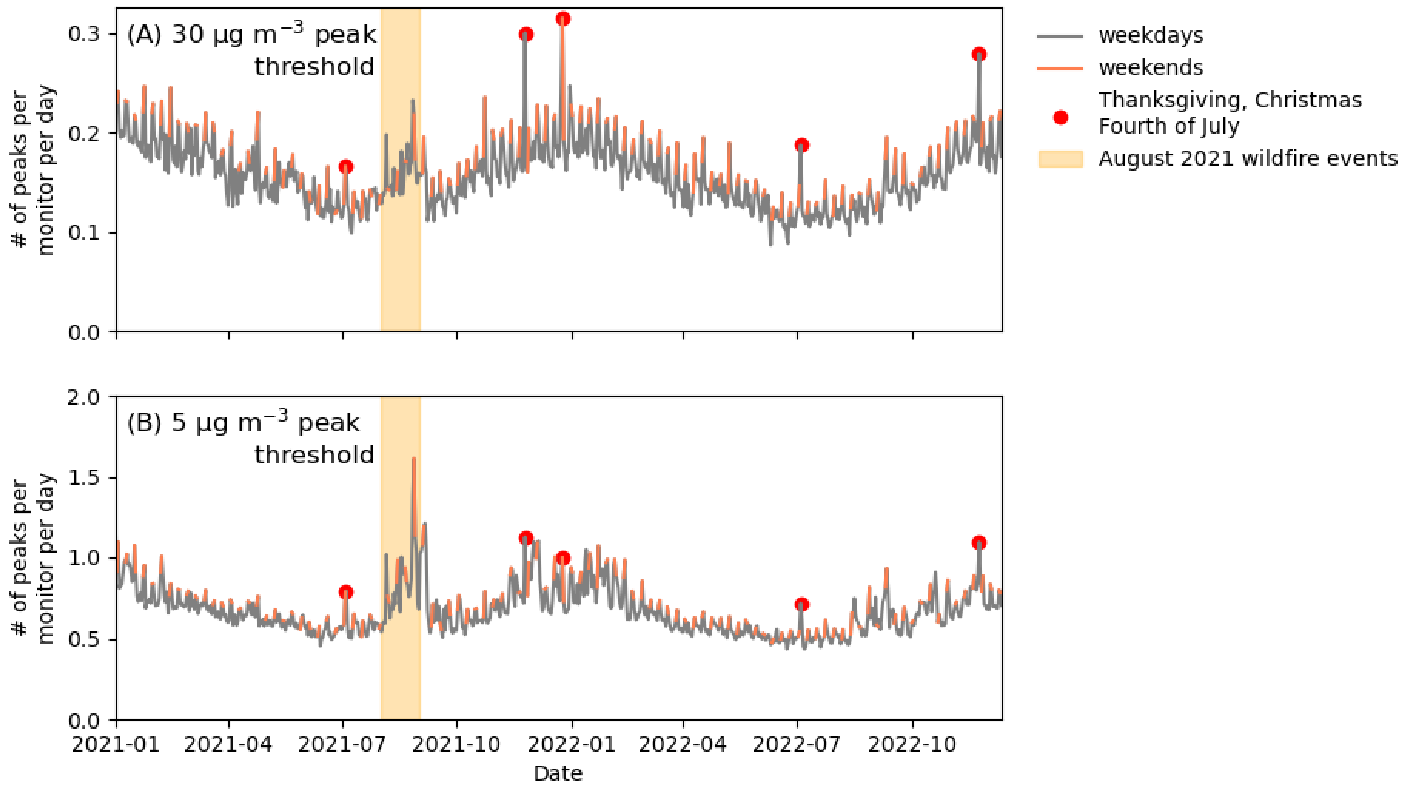

Figure S8. The number of peaks per monitor per day (peak threshold = 30 µg m^{-3}, panel A; peak threshold = 5 µg m^{-3}, panel B) are plotted over time. Weekdays are displayed in gray and weekends are displayed in light red. Major holidays with traditional gatherings (Thanksgiving, Christmas, Fourth of July) are highlighted with red circles.

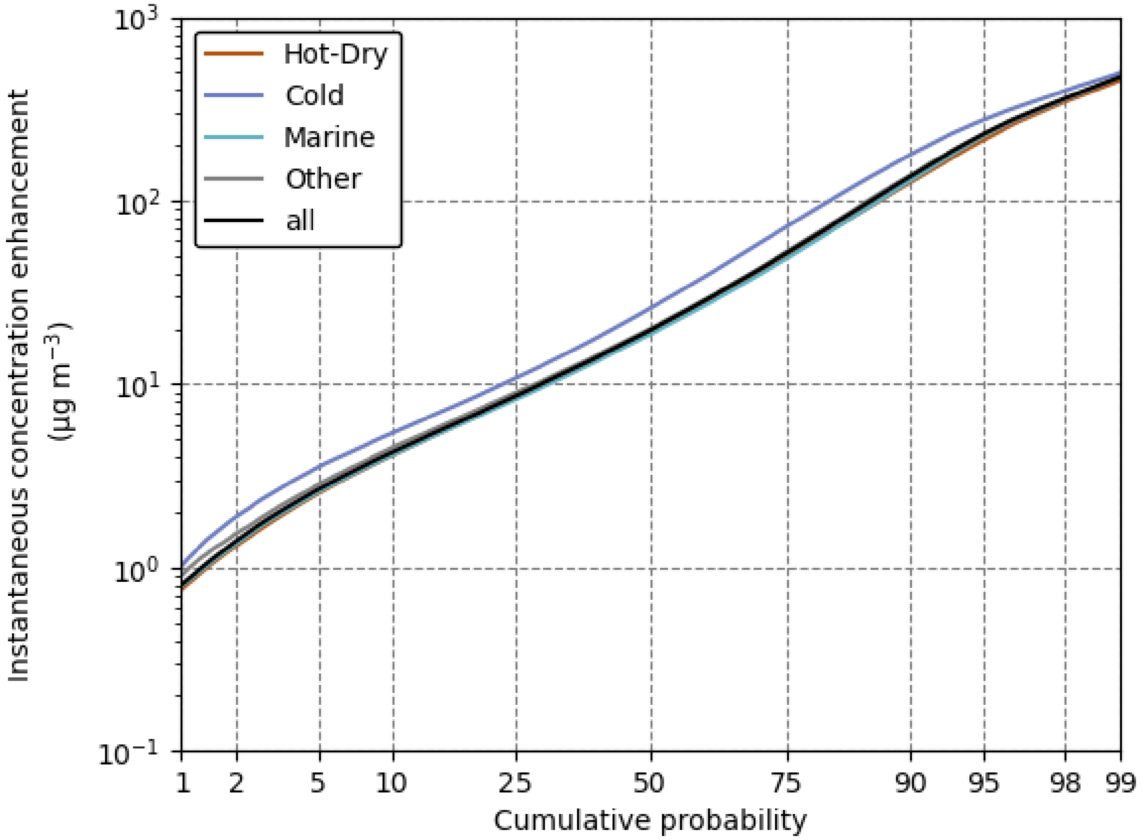

Figure S9. Distribution of instantaneous concentration enhancements in units of mass concentration (µg m^{-3}) over all residences (*n* = 3977 residences) for emission events with peak prominences greater than 5 µg m^{-3}. Approximately 1.31 million emission events with peak prominences greater than 5 µg m^{-3} were extracted from 5338 monitor-years of indoor data. Considering all climate zones, the arithmetic mean and arithmetic standard deviation are 54 µg m^{-3} and 153 µg m^{-3}, respectively, and the geometric mean and geometric standard deviation are 21.2 µg m^{-3} and 4.2, respectively.

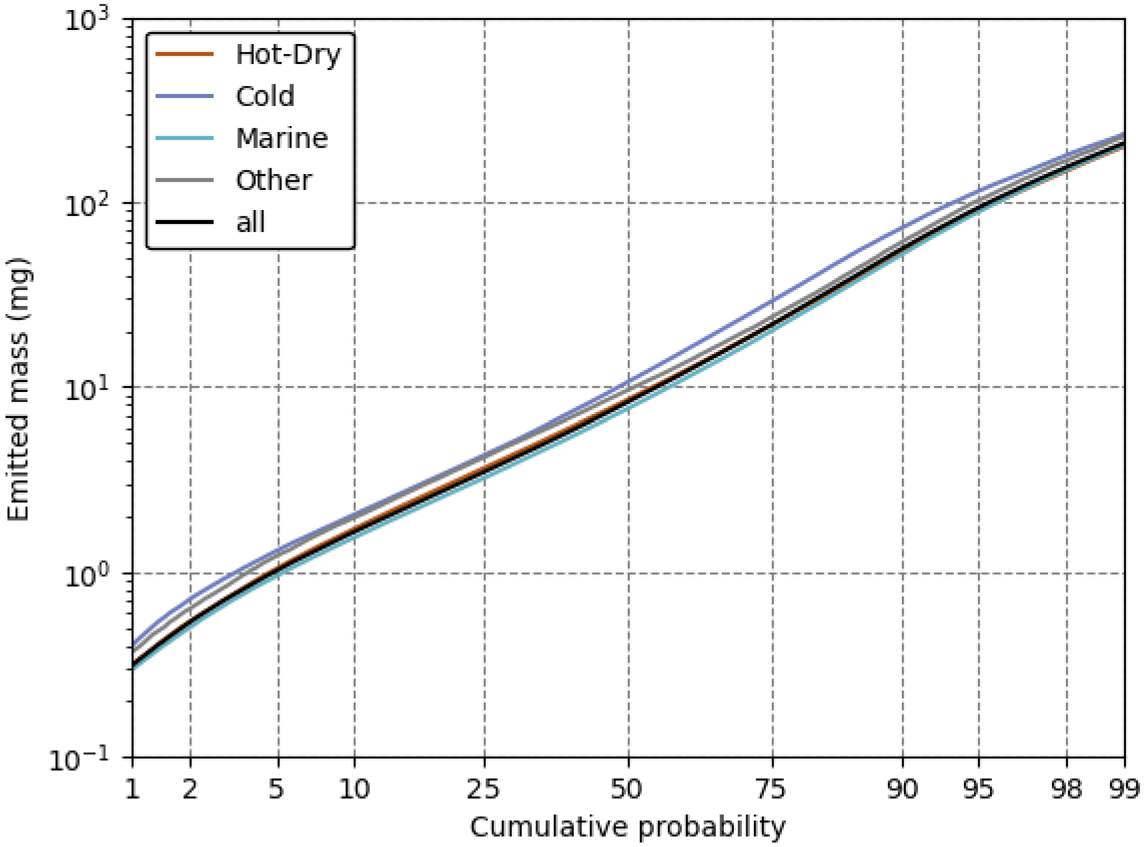

Figure S10. Distribution of emission masses from episodic events (units = mg) over single family residences (*n* = 3423 single family residences). Approximately 1.10 million emission events with peak prominences greater than 5 μg m^{-3} were extracted from 4630 monitor-years of indoor data. Arithmetic mean and arithmetic standard deviation are 23 and 46 mg, respectively, and the geometric mean and geometric standard deviation are 8.7 mg and 4.3, respectively.

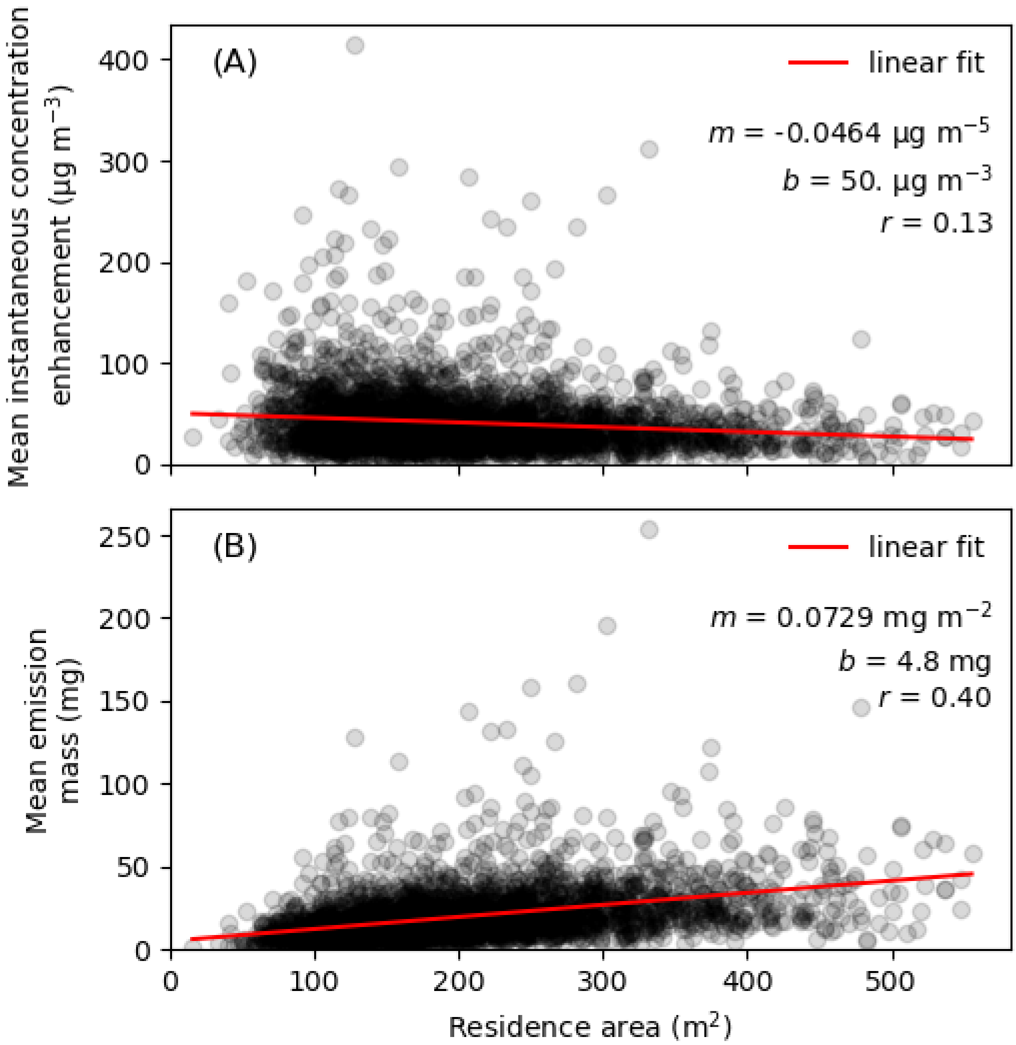

Figure S11. The relationship between instantaneous concentration enhancements (panel A – µg m^{-3}) or emission mass (panel B – mg) and residence area over single family residences (*n* = 3423 single family residences).

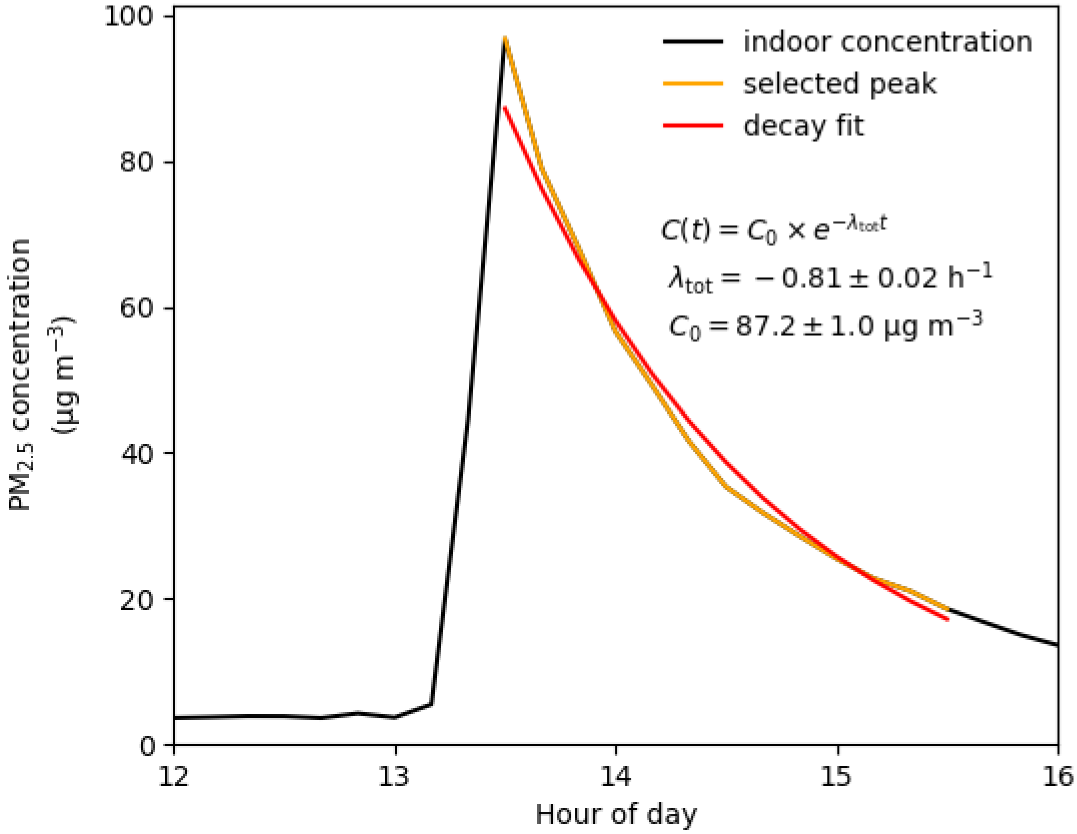

Figure S12. The decay period (orange) of a well-behaved indoor emission event in $PM_{2.5}$ (black) is fit to an exponential function (red). The exponential decay constant is interpreted as the particle loss-rate coefficient.

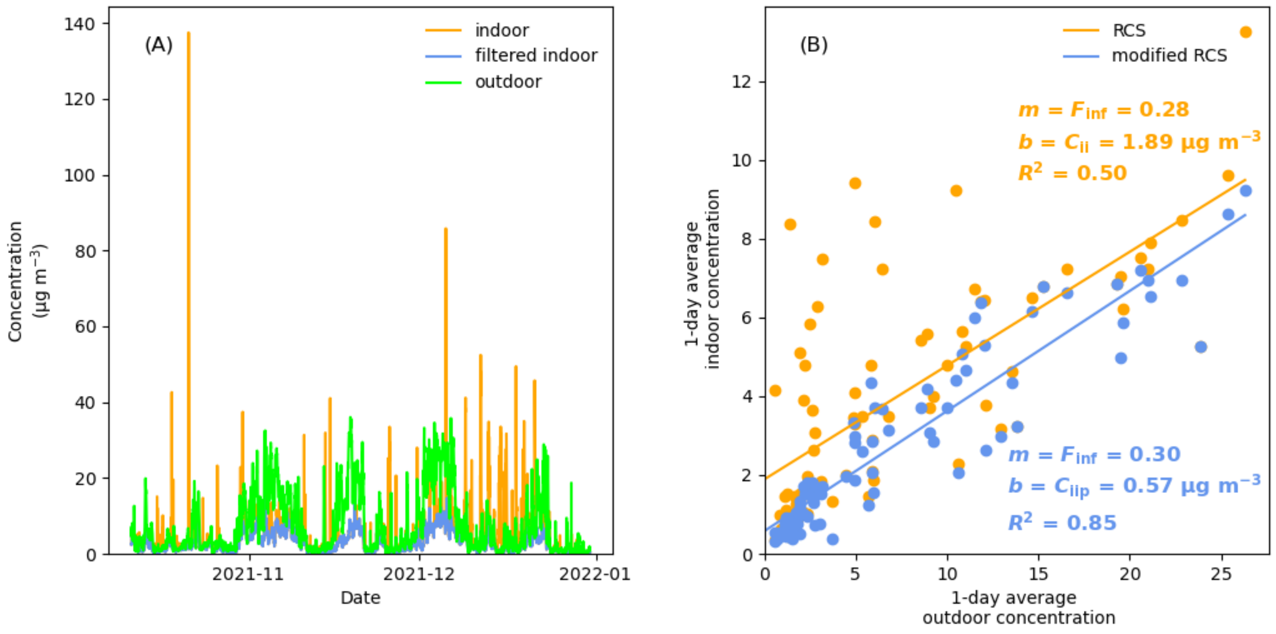

Figure S13. In panel A, a time-series of indoor (orange), excised indoor (blue), and outdoor (lime) $PM_{2.5}$ concentrations are displayed over two months. In panel B, RCS and modified RCS analysis is performed by regressing daily averages of indoor (orange) and excised indoor (blue) $PM_{2.5}$ concentrations against outdoor $PM_{2.5}$ daily averages. Resulting fit parameters are displayed.

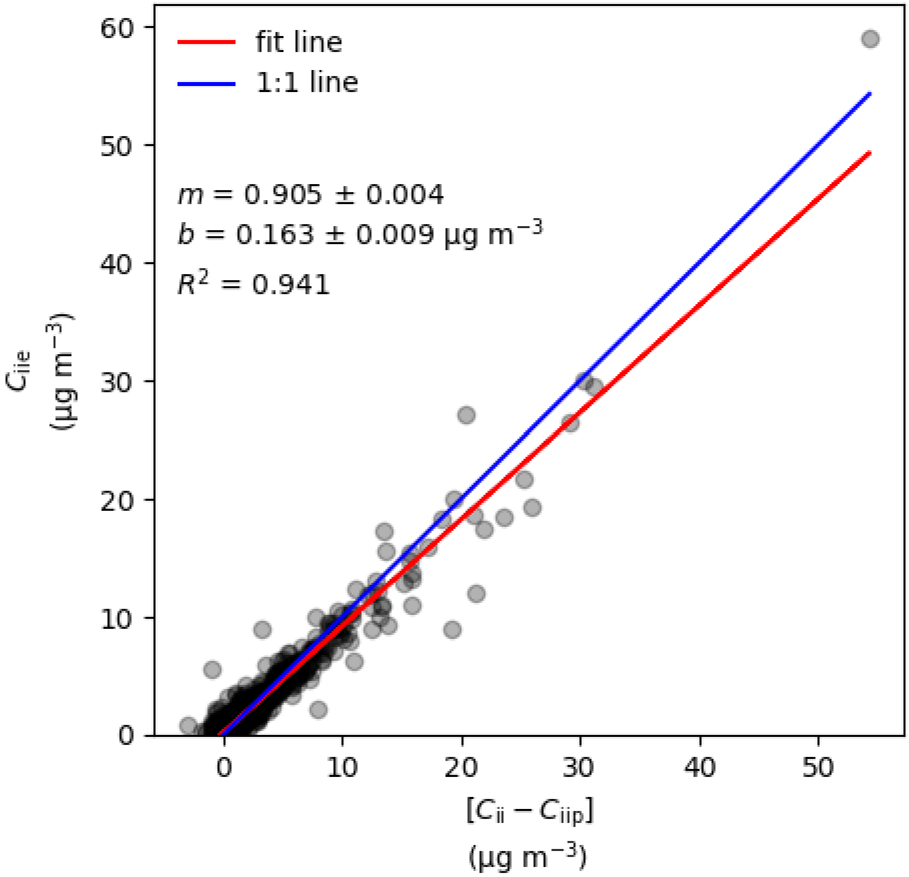

Figure S14. A scatter plot comparing the average indoor episodic concentration against the indoor concentration of indoor origin (C_{ii} via RCS) minus the indoor concentration attributable to persistent indoor emissions (C_{iip}via modified RCS). A linear regression (red) with fitted parameters and a 1:1 line (blue) are also displayed.

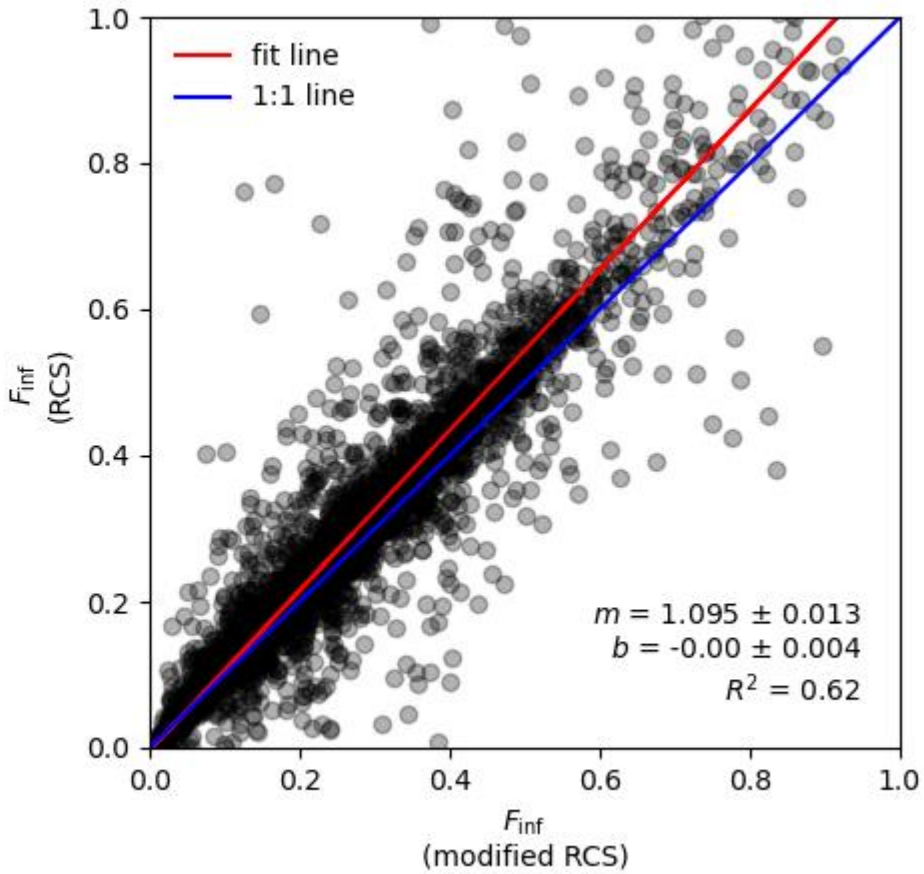

Figure S15. A scatter plot comparing the infiltration factor, F_{inf}, obtained via modified RCS, against the infiltration factor, F_{inf}, obtained via the original RCS method (8). A linear regression (red) with fitted parameters and a 1:1 line (blue) are also displayed. Non-physical infiltration factor values (RCS: 72 values, modified RCS: 1 value) are not displayed.

Table S1. Location of monitors in climate zones and states.

climate zone	US state	% of total	number
Total *n* = 3977	CA	74	2924
	WA	7	259
	OR	4	162
	CO	4	144
	UT	4	141
	other	9	347
Hot-Dry *n* = 787	CA	99	781
	other	1	6
Cold *n* = 625	UT	23	141
	CO	22	137
	NV	13	84
	CA	9	58
	other	33	205
Marine *n* = 2360	CA	85	2014
	WA	9	215
	OR	6	131
Other *n* = 205	CA	35	71
	NY	16	33
	TX	15	31
	NC	6	12
	other	28	58

Table S2. Key summary statistics and major measurement results during the spring season (March, April, May).[a]

		time series analysis				modified RCS analysis			traditional RCS analysis		indoor generated fractions		
		$\bar{\lambda}_{tot}$	C_i	C_{iie}	C_o	C_{io}	C_{iip}	F_{inf}	C_{ii}	F_{inf}	$\frac{C_{iie}}{C_i}$	$\frac{C_{iip}}{C_i}$	$\frac{C_{iip}+C_{iie}}{C_i}$
	**units**	h^{-1}	μg m^{-3}	μg m^{-3}	μg m^{-3}	μg m^{-3}	μg m^{-3}	none	μg m^{-3}	none	none	none	none
Total	10th quantile	0.66	0.83	0.08	2.12	0.32	-0.28	0.11	0.04	0.04	0.06	-0.13	0.02
n = 3977	**median**	**1.42**	**1.99**	**0.50**	**2.98**	**1.25**	**0.09**	**0.42**	**0.79**	**0.35**	**0.28**	**0.05**	**0.39**
	90th quantile	2.77	4.23	2.27	4.59	2.31	0.57	0.71	3.26	0.69	0.65	0.33	0.78
	**mean**	**1.61**	**2.49**	**1.06**	**3.25**	**1.31**	**0.12**	**0.41**	**1.41**	**0.36**	**0.32**	**0.07**	**0.38**
	st. dev.	0.95	2.35	2.09	1.19	0.80	0.37	0.22	2.61	0.46	0.22	0.21	0.30
Marine	10th quantile	0.70	0.94	0.07	2.42	0.51	-0.33	0.16	0.00	0.08	0.06	-0.16	-0.02
n = 2360	**median**	**1.45**	**2.01**	**0.45**	**3.07**	**1.40**	**0.03**	**0.46**	**0.71**	**0.38**	**0.25**	**0.02**	**0.32**
	90th quantile	2.81	3.83	1.91	3.96	2.28	0.46	0.72	2.88	0.70	0.58	0.27	0.68
	**mean**	**1.66**	**2.36**	**0.90**	**3.16**	**1.40**	**0.05**	**0.45**	**1.21**	**0.39**	**0.29**	**0.03**	**0.32**
	st. dev.	0.95	1.83	1.59	0.65	0.67	0.34	0.21	2.26	0.41	0.20	0.20	0.28
Hot-Dry	10th quantile	0.60	0.89	0.08	2.29	0.39	-0.18	0.12	0.12	0.04	0.06	-0.09	0.10
n = 787	**median**	**1.39**	**2.13**	**0.50**	**3.17**	**1.29**	**0.16**	**0.41**	**0.86**	**0.35**	**0.26**	**0.08**	**0.39**
	90th quantile	2.52	4.87	2.21	6.28	2.72	0.66	0.67	3.26	0.65	0.61	0.32	0.72
	**mean**	**1.54**	**2.72**	**1.05**	**3.76**	**1.47**	**0.20**	**0.40**	**1.47**	**0.35**	**0.30**	**0.09**	**0.39**
	st. dev.	0.93	2.41	2.00	1.60	0.96	0.41	0.21	2.52	0.42	0.21	0.19	0.26
Cold	10th quantile	0.65	0.59	0.10	1.36	0.12	-0.07	0.05	0.17	-0.14	0.12	-0.03	0.30
n = 625	**median**	**1.35**	**1.62**	**0.70**	**2.12**	**0.50**	**0.25**	**0.22**	**1.12**	**0.18**	**0.50**	**0.14**	**0.73**
	90th quantile	2.83	5.42	3.95	3.98	1.32	0.64	0.61	4.83	0.74	0.81	0.44	0.92
	**mean**	**1.58**	**2.69**	**1.77**	**2.45**	**0.67**	**0.25**	**0.28**	**2.16**	**0.27**	**0.49**	**0.17**	**0.66**
	st. dev.	0.94	3.87	3.63	1.24	0.69	0.33	0.22	3.90	0.68	0.25	0.22	0.27
Other	10th quantile	0.49	0.69	0.07	1.79	0.30	-0.11	0.09	0.08	0.05	0.06	-0.07	0.08
n = 205	**median**	**1.29**	**2.24**	**0.48**	**4.75**	**1.20**	**0.21**	**0.29**	**0.92**	**0.27**	**0.24**	**0.11**	**0.44**
	90th quantile	2.79	5.44	2.64	7.05	2.42	0.83	0.63	3.34	0.70	0.62	0.36	0.72
	**mean**	**1.48**	**2.71**	**1.06**	**4.48**	**1.35**	**0.27**	**0.34**	**1.41**	**0.31**	**0.30**	**0.12**	**0.41**
	st. dev.	0.94	2.17	1.63	2.27	1.01	0.44	0.21	1.89	0.37	0.22	0.23	0.29

[a] Concentration values (C_i, C_{iie}, C_o, C_{ii}, C_{iip}) are reported in units of μg m^{-3}. The infiltration factor, F_{inf}, and the fractions of indoor $PM_{2.5}$ of episodic indoor origin, persistent indoor origin, and total indoor origin, that is C_{iie}/C_i, C_{iip}/C_i, and $(C_{iie}+C_{iip})/C_i$ are unitless. The mean of well-behaved loss-rate coefficients ($\bar{\lambda}_{tot}$) is reported in units of h^{-1}. Monitors that did not report data during this season were excluded from analysis.

Table S3. Key summary statistics and major measurement results during the summer season (June, July, August).[a]

		time series analysis				modified RCS analysis			traditional RCS analysis		indoor generated fractions		
		$\bar{\lambda}_{tot}$	C_i	C_{iie}	C_o	C_{io}	C_{iip}	F_{inf}	C_{ii}	F_{inf}	$\frac{C_{iie}}{C_i}$	$\frac{C_{iip}}{C_i}$	$\frac{C_{iip}+C_{iie}}{C_i}$
	**units**	h^{-1}	µg m^{-3}	µg m^{-3}	µg m^{-3}	µg m^{-3}	µg m^{-3}	none	µg m^{-3}	none	none	none	none
Total	10th quantile	0.70	1.27	0.12	3.63	0.44	-0.30	0.08	-0.26	0.08	0.06	-0.10	0.04
n = 3977	**median**	**1.54**	**2.83**	**0.45**	**4.96**	**1.54**	**0.44**	**0.31**	**0.94**	**0.33**	**0.17**	**0.18**	**0.42**
	90th quantile	2.94	5.17	1.64	8.66	3.41	1.44	0.68	2.72	0.79	0.45	0.52	0.77
	**mean**	**1.72**	**3.23**	**0.86**	**5.83**	**1.84**	**0.49**	**0.35**	**1.20**	**0.40**	**0.22**	**0.18**	**0.40**
	st. dev.	0.96	2.36	1.58	3.67	1.46	0.77	0.23	2.00	0.34	0.17	0.27	0.31
Marine	10th quantile	0.75	1.38	0.11	3.78	0.56	-0.30	0.11	-0.25	0.11	0.06	-0.11	0.02
n = 2360	**median**	**1.59**	**2.80**	**0.40**	**4.74**	**1.65**	**0.41**	**0.35**	**0.88**	**0.37**	**0.16**	**0.17**	**0.38**
	90th quantile	2.97	4.43	1.37	6.24	3.15	1.37	0.69	2.45	0.79	0.39	0.51	0.72
	**mean**	**1.76**	**2.98**	**0.70**	**5.01**	**1.79**	**0.46**	**0.38**	**1.07**	**0.42**	**0.20**	**0.17**	**0.37**
	st. dev.	0.96	1.72	1.25	1.61	1.05	0.73	0.22	1.74	0.30	0.15	0.28	0.30
Hot-Dry	10th quantile	0.62	1.31	0.13	3.91	0.39	-0.19	0.06	-0.26	0.06	0.06	-0.07	0.06
n = 787	**median**	**1.49**	**2.95**	**0.50**	**6.37**	**1.43**	**0.58**	**0.25**	**1.02**	**0.29**	**0.17**	**0.22**	**0.48**
	90th quantile	2.79	6.35	1.59	9.40	4.57	1.62	0.65	2.91	0.74	0.43	0.55	0.80
	**mean**	**1.67**	**3.54**	**0.86**	**6.91**	**2.04**	**0.61**	**0.31**	**1.31**	**0.36**	**0.22**	**0.22**	**0.44**
	st. dev.	0.97	2.38	1.42	4.36	1.86	0.84	0.23	1.92	0.31	0.16	0.28	0.31
Cold	10th quantile	0.65	1.10	0.14	2.68	0.29	-0.31	0.05	-0.13	0.05	0.08	-0.08	0.07
n = 625	**median**	**1.47**	**2.79**	**0.68**	**5.85**	**1.25**	**0.38**	**0.22**	**1.10**	**0.24**	**0.26**	**0.17**	**0.51**
	90th quantile	2.91	7.20	2.86	13.48	4.30	1.48	0.69	3.61	0.83	0.62	0.48	0.83
	**mean**	**1.65**	**3.73**	**1.40**	**7.05**	**1.82**	**0.46**	**0.30**	**1.62**	**0.37**	**0.31**	**0.18**	**0.49**
	st. dev.	0.91	3.41	2.33	5.82	2.00	0.78	0.25	2.92	0.50	0.21	0.24	0.30
Other	10th quantile	0.50	0.95	0.09	3.10	0.33	-0.48	0.05	-0.43	0.05	0.06	-0.14	0.00
n = 205	**median**	**1.43**	**2.56**	**0.50**	**6.71**	**1.33**	**0.37**	**0.20**	**0.72**	**0.27**	**0.20**	**0.19**	**0.48**
	90th quantile	2.89	6.76	2.18	10.61	4.11	1.52	0.56	2.72	0.77	0.50	0.52	0.82
	**mean**	**1.61**	**3.40**	**1.04**	**7.46**	**1.78**	**0.46**	**0.27**	**0.98**	**0.35**	**0.25**	**0.19**	**0.43**
	st. dev.	1.03	4.13	2.35	6.30	1.84	0.83	0.22	1.61	0.33	0.17	0.26	0.32

[a] Concentration values (C_i, C_{iie}, C_o, C_{ii}, C_{iip}) are reported in units of µg m^{-3}. The infiltration factor, F_{inf}, and the fractions of indoor $PM_{2.5}$ of episodic indoor origin, persistent indoor origin, and total indoor origin, that is C_{iie}/C_i, C_{iip}/C_i, and $(C_{iie} + C_{iip})/C_i$ are unitless. The mean of well-behaved loss-rate coefficients ($\bar{\lambda}_{tot}$) is reported in units of h^{-1}. Monitors that did not report data during this season were excluded from analysis.

Table S4. Key summary statistics and major measurement results during the fall season (September, October, November).[a]

		time series analysis				modified RCS analysis			traditional RCS analysis		indoor generated fractions		
		$\bar{\lambda}_{\mathrm{tot}}$	C_{i}	C_{iie}	C_{o}	C_{io}	C_{iip}	F_{inf}	C_{ii}	F_{inf}	$\frac{C_{\mathrm{iie}}}{C_i}$	$\frac{C_{\mathrm{iip}}}{C_i}$	$\frac{C_{\mathrm{iip}}+C_{\mathrm{iie}}}{C_i}$
	**units**	h^{-1}	μg m^{-3}	μg m^{-3}	μg m^{-3}	μg m^{-3}	μg m^{-3}	none	μg m^{-3}	none	none	none	none
Total	10th quantile	0.58	1.36	0.13	4.81	0.56	-0.23	0.08	0.10	0.08	0.07	-0.07	0.10
n = 3977	**median**	**1.26**	**3.17**	**0.61**	**6.49**	**1.84**	**0.43**	**0.28**	**1.08**	**0.28**	**0.21**	**0.15**	**0.41**
	90th quantile	2.48	6.03	2.38	10.01	3.69	1.18	0.57	3.29	0.57	0.51	0.40	0.72
	**mean**	**1.44**	**3.69**	**1.19**	**7.05**	**2.06**	**0.44**	**0.31**	**1.57**	**0.31**	**0.25**	**0.15**	**0.40**
	st. dev.	0.88	3.23	2.66	2.72	1.40	0.67	0.19	2.38	0.22	0.18	0.20	0.25
Marine	10th quantile	0.60	1.49	0.13	5.30	0.70	-0.20	0.10	0.12	0.10	0.06	-0.06	0.08
n = 2360	**median**	**1.26**	**3.21**	**0.55**	**6.34**	**2.00**	**0.42**	**0.30**	**1.02**	**0.30**	**0.19**	**0.14**	**0.38**
	90th quantile	2.47	5.63	2.15	9.30	3.60	1.09	0.57	3.01	0.56	0.44	0.38	0.66
	**mean**	**1.45**	**3.54**	**0.97**	**6.99**	**2.14**	**0.43**	**0.32**	**1.41**	**0.32**	**0.22**	**0.15**	**0.37**
	st. dev.	0.89	2.32	1.63	2.63	1.32	0.63	0.18	1.93	0.20	0.15	0.19	0.24
Hot-Dry	10th quantile	0.58	1.41	0.15	5.70	0.56	-0.06	0.07	0.25	0.07	0.07	-0.02	0.15
n = 787	**median**	**1.25**	**3.40**	**0.63**	**7.64**	**1.88**	**0.56**	**0.25**	**1.22**	**0.25**	**0.20**	**0.18**	**0.43**
	90th quantile	2.47	6.36	2.17	10.35	3.93	1.38	0.53	3.41	0.53	0.47	0.44	0.70
	**mean**	**1.43**	**3.92**	**1.17**	**7.97**	**2.13**	**0.61**	**0.28**	**1.78**	**0.28**	**0.24**	**0.20**	**0.43**
	st. dev.	0.86	2.89	2.15	2.26	1.42	0.68	0.18	2.76	0.20	0.16	0.20	0.23
Cold	10th quantile	0.58	1.12	0.15	3.07	0.32	-0.51	0.06	-0.01	0.05	0.11	-0.16	0.05
n = 625	**median**	**1.26**	**2.67**	**0.88**	**5.48**	**1.24**	**0.28**	**0.25**	**1.16**	**0.23**	**0.35**	**0.11**	**0.53**
	90th quantile	2.48	7.46	3.35	10.43	3.69	1.02	0.64	3.92	0.63	0.66	0.40	0.82
	**mean**	**1.44**	**3.65**	**1.66**	**6.29**	**1.72**	**0.28**	**0.29**	**1.87**	**0.30**	**0.37**	**0.11**	**0.48**
	st. dev.	0.84	3.58	2.88	3.19	1.56	0.71	0.22	3.25	0.29	0.20	0.22	0.30
Other	10th quantile	0.52	1.03	0.13	3.26	0.39	-0.24	0.06	-0.07	0.06	0.09	-0.08	0.12
n = 205	**median**	**1.24**	**2.94**	**0.69**	**6.15**	**1.38**	**0.38**	**0.24**	**1.07**	**0.24**	**0.26**	**0.16**	**0.49**
	90th quantile	2.52	7.41	3.66	10.24	3.53	1.40	0.58	4.10	0.60	0.61	0.51	0.83
	**mean**	**1.41**	**4.59**	**2.33**	**6.56**	**1.75**	**0.47**	**0.29**	**1.72**	**0.29**	**0.31**	**0.17**	**0.47**
	st. dev.	0.95	8.28	7.84	2.78	1.54	0.73	0.22	2.34	0.25	0.21	0.23	0.28

[a] Concentration values (C_{i}, C_{iie}, C_{o}, C_{ii}, C_{iip}) are reported in units of μg m^{-3}. The infiltration factor, F_{inf}, and the fractions of indoor $PM_{2.5}$ of episodic indoor origin, persistent indoor origin, and total indoor origin, that is $C_{\mathrm{iie}}/C_{\mathrm{i}}$, $C_{\mathrm{iip}}/C_{\mathrm{i}}$, and $(C_{\mathrm{iie}} + C_{\mathrm{iip}})/C_{\mathrm{i}}$ are unitless. The mean of well-behaved loss-rate coefficients ($\bar{\lambda}_{\mathrm{tot}}$) is reported in units of h^{-1}. Monitors that did not report data during this season were excluded from analysis.

Table S5. Key summary statistics and major measurement results during the winter season (December, January, February).[a]

		time series analysis				modified RCS analysis			traditional RCS analysis		indoor generated fractions		
		$\bar{\lambda}_{tot}$	C_i	C_{iie}	C_o	C_{io}	C_{iip}	F_{inf}	C_{ii}	F_{inf}	$\frac{C_{iie}}{C_i}$	$\frac{C_{iip}}{C_i}$	$\frac{C_{iip}+C_{iie}}{C_i}$
	**units**	h^{-1}	μg m^{-3}	μg m^{-3}	μg m^{-3}	μg m^{-3}	μg m^{-3}	none	μg m^{-3}	none	none	none	none
Total	10th quantile	0.59	0.89	0.11	2.90	0.37	0.03	0.07	0.26	0.04	0.08	0.01	0.22
n = 3977	**median**	**1.28**	**2.48**	**0.67**	**6.51**	**1.26**	**0.38**	**0.19**	**1.10**	**0.18**	**0.30**	**0.16**	**0.50**
	90th quantile	2.64	5.71	2.99	9.26	2.74	0.87	0.39	3.60	0.40	0.66	0.36	0.78
	**mean**	**1.50**	**3.21**	**1.43**	**6.54**	**1.46**	**0.41**	**0.22**	**1.82**	**0.22**	**0.34**	**0.17**	**0.50**
	st. dev.	0.99	3.56	3.10	2.85	1.06	0.40	0.14	2.93	0.50	0.22	0.15	0.21
Marine	10th quantile	0.61	1.01	0.10	4.02	0.52	0.05	0.08	0.27	0.07	0.08	0.02	0.22
n = 2360	**median**	**1.25**	**2.51**	**0.64**	**6.55**	**1.32**	**0.40**	**0.20**	**1.06**	**0.20**	**0.27**	**0.16**	**0.48**
	90th quantile	2.59	5.21	2.53	8.85	2.68	0.86	0.38	3.31	0.40	0.58	0.35	0.72
	**mean**	**1.49**	**3.05**	**1.23**	**6.51**	**1.49**	**0.42**	**0.22**	**1.63**	**0.22**	**0.30**	**0.17**	**0.47**
	st. dev.	0.99	2.86	2.42	1.88	0.90	0.37	0.13	2.27	0.16	0.19	0.14	0.20
Hot-Dry	10th quantile	0.57	1.02	0.13	3.76	0.47	0.05	0.06	0.30	0.05	0.09	0.01	0.22
n = 787	**median**	**1.29**	**2.77**	**0.67**	**7.99**	**1.46**	**0.44**	**0.18**	**1.17**	**0.17**	**0.27**	**0.16**	**0.47**
	90th quantile	2.51	6.47	2.87	14.13	3.55	0.97	0.37	4.13	0.36	0.62	0.36	0.74
	**mean**	**1.46**	**3.61**	**1.47**	**8.58**	**1.78**	**0.47**	**0.20**	**2.04**	**0.19**	**0.31**	**0.17**	**0.47**
	st. dev.	0.99	3.77	3.08	4.01	1.37	0.42	0.13	3.97	0.19	0.20	0.14	0.21
Cold	10th quantile	0.65	0.58	0.11	1.65	0.16	0.00	0.05	0.21	-0.03	0.13	0.00	0.33
n = 625	**median**	**1.45**	**1.90**	**0.79**	**3.94**	**0.52**	**0.25**	**0.14**	**1.21**	**0.09**	**0.51**	**0.14**	**0.71**
	90th quantile	2.76	6.43	4.23	7.66	1.75	0.63	0.43	5.73	0.45	0.81	0.37	0.89
	**mean**	**1.62**	**3.02**	**1.94**	**4.31**	**0.79**	**0.28**	**0.20**	**2.37**	**0.22**	**0.49**	**0.17**	**0.66**
	st. dev.	0.94	4.17	3.81	2.27	0.87	0.36	0.19	3.78	1.30	0.26	0.16	0.22
Other	10th quantile	0.47	0.69	0.07	2.38	0.21	-0.25	0.05	0.14	0.04	0.06	-0.06	0.17
n = 205	**median**	**1.17**	**2.92**	**0.69**	**6.27**	**1.34**	**0.35**	**0.22**	**1.14**	**0.20**	**0.31**	**0.16**	**0.48**
	90th quantile	2.91	6.53	4.31	8.83	2.71	1.02	0.53	4.80	0.57	0.72	0.42	0.81
	**mean**	**1.45**	**4.03**	**2.12**	**5.93**	**1.50**	**0.40**	**0.26**	**1.81**	**0.27**	**0.35**	**0.17**	**0.49**
	st. dev.	1.05	6.55	6.08	2.87	1.24	0.64	0.20	2.20	0.34	0.25	0.20	0.24

[a] Concentration values (C_i, C_{iie}, C_o, C_{ii}, C_{iip}) are reported in units of μg m^{-3}. The infiltration factor, F_{inf}, and the fractions of indoor $PM_{2.5}$ of episodic indoor origin, persistent indoor origin, and total indoor origin, that is C_{iie}/C_i, C_{iip}/C_i, and $(C_{iie}+C_{iip})/C_i$ are unitless. The mean of well-behaved loss-rate coefficients ($\bar{\lambda}_{tot}$) is reported in units of h^{-1}. Monitors that did not report data during this season were excluded from analysis.

Table S6. Key summary statistics for values across climate zones in the western and eastern regions of the United States.[a]

		time series analysis				modified RCS analysis			traditional RCS analysis		indoor generated fractions		
		$\bar{\lambda}_{tot}$	C_i	C_{iie}	C_o	C_{io}	C_{iip}	F_{inf}	C_{ii}	F_{inf}	$\frac{C_{iie}}{C_i}$	$\frac{C_{iip}}{C_i}$	$\frac{C_{iip}+C_{iie}}{C_i}$
n **=**	**region**												
	**mean**	h^{-1}	μg m^{-3}	μg m^{-3}	μg m^{-3}	μg m^{-3}	μg m^{-3}	none	μg m^{-3}	none	none	none	none
2360	Marine (West)	**1.56**	**3.12**	**0.98**	**5.78**	**1.61**	**0.52**	**0.29**	**1.52**	**0.29**	**0.25**	**0.2**	**0.46**
86	Mixed-Humid (East)	1.47	5.51	3.19	6.12	1.82	0.41	0.32	2.57	0.38	0.31	0.14	0.43
538	Cold (West)	**1.55**	**3.32**	**1.64**	**5.38**	**1.33**	**0.36**	**0.26**	**2.01**	**0.27**	**0.41**	**0.14**	**0.54**
87	Cold (East)	1.44	4.12	2.05	5.74	1.93	0.16	0.34	2.43	0.31	0.34	0.11	0.45
787	Hot-Dry (West)	**1.51**	**3.56**	**1.15**	**7**	**1.72**	**0.68**	**0.26**	**1.89**	**0.26**	**0.26**	**0.23**	**0.5**
38	Hot-Humid (East)	1.36	3.04	0.91	6.05	1.74	0.36	0.29	1.35	0.29	0.26	0.14	0.4
	**coefficient of variation**	none	none	none	none	none	none	none	none	none	none	none	none
2360	Marine (West)	**55%**	**70%**	**173%**	**32%**	**62%**	**101%**	**58%**	**120%**	**62%**	**64%**	**94%**	**49%**
86	Mixed-Humid (East)	67%	194%	320%	27%	66%	131%	67%	253%	207%	69%	140%	61%
538	Cold (West)	**50%**	**91%**	**149%**	**49%**	**91%**	**175%**	**76%**	**147%**	**94%**	**49%**	**155%**	**53%**
87	Cold (East)	55%	124%	224%	27%	82%	386%	75%	199%	120%	68%	173%	60%
787	Hot-Dry (West)	**55%**	**75%**	**177%**	**30%**	**70%**	**91%**	**67%**	**136%**	**79%**	**63%**	**81%**	**44%**
38	Hot-Humid (East)	77%	63%	121%	24%	73%	159%	66%	98%	74%	71%	130%	60%

[a] Concentration values (C_i, C_{iie}, C_o, C_{ii}, C_{iip}) are reported in units of μg m^{-3}. The infiltration factor, F_{inf}, and the fractions of indoor $PM_{2.5}$ of episodic indoor origin, persistent indoor origin, and total indoor origin, that is C_{iie}/C_i, C_{iip}/C_i, and $(C_{iie} + C_{iip})/C_i$ are unitless. The mean of well-behaved loss-rate coefficients ($\bar{\lambda}_{tot}$) is reported in units of h^{-1}. Regions correspond Building America climate zone designations subdivided by western states ("West": MT to NM and westward) and states to the east ("East": remainder of the contiguous US).

Table S7. Key summary statistics and major measurement results for residences binned by population density.[a]

		time series analysis				modified RCS analysis			traditional RCS analysis		indoor generated fractions		
**Population density**		$\bar{\lambda}_{tot}$	C_i	C_{iie}	C_o	C_{io}	C_{iip}	F_{inf}	C_{ii}	F_{inf}	$\frac{C_{iie}}{C_i}$	$\frac{C_{iip}}{C_i}$	$\frac{C_{iip}+C_{iie}}{C_i}$
	**units**	h^{-1}	µg m^{-3}	µg m^{-3}	µg m^{-3}	µg m^{-3}	µg m^{-3}	none	µg m^{-3}	none	none	none	none
0–100	10th quantile	0.68	1.28	0.20	3.21	0.38	-0.49	0.06	-0.06	0.07	0.12	-0.14	0.07
persons	**median**	**1.32**	**2.87**	**0.78**	**5.68**	**1.33**	**0.44**	**0.24**	**1.17**	**0.26**	**0.29**	**0.17**	**0.54**
km^{-2}	90th quantile	2.43	6.85	3.36	9.45	3.34	1.29	0.65	3.77	0.70	0.60	0.46	0.83
n = 498	**mean**	**1.47**	**3.51**	**1.40**	**6.24**	**1.68**	**0.42**	**0.30**	**1.70**	**0.32**	**0.33**	**0.16**	**0.49**
	st. dev.	0.75	2.42	1.82	3.02	1.38	0.75	0.22	2.22	0.28	0.19	0.24	0.29
100–500	10th quantile	0.66	1.21	0.14	3.90	0.41	-0.17	0.07	0.21	0.07	0.09	-0.06	0.14
persons	**median**	**1.30**	**2.57**	**0.60**	**5.05**	**1.24**	**0.45**	**0.25**	**1.04**	**0.26**	**0.25**	**0.18**	**0.49**
km^{-2}	90th quantile	2.41	4.78	2.05	7.99	2.76	1.06	0.54	2.72	0.55	0.56	0.45	0.78
n = 834	**mean**	**1.46**	**3.05**	**1.15**	**5.58**	**1.47**	**0.44**	**0.28**	**1.60**	**0.29**	**0.29**	**0.18**	**0.47**
	st. dev.	0.80	2.79	2.36	1.99	1.00	0.53	0.19	2.66	0.20	0.18	0.20	0.25
500–1000	10th quantile	0.69	1.20	0.15	3.49	0.43	-0.03	0.09	0.30	0.08	0.09	-0.01	0.17
persons	**median**	**1.32**	**2.63**	**0.61**	**5.27**	**1.29**	**0.51**	**0.25**	**1.18**	**0.26**	**0.26**	**0.19**	**0.52**
km^{-2}	90th quantile	2.54	5.30	2.44	7.22	2.74	1.05	0.46	3.36	0.47	0.59	0.42	0.76
n = 471	**mean**	**1.52**	**3.09**	**1.12**	**5.47**	**1.47**	**0.49**	**0.27**	**1.68**	**0.27**	**0.30**	**0.19**	**0.50**
	st. dev.	0.84	2.24	1.74	1.77	1.06	0.48	0.16	2.13	0.17	0.19	0.18	0.23
1000–2000	10th quantile	0.72	1.28	0.15	4.50	0.49	-0.01	0.08	0.29	0.08	0.09	0.00	0.18
persons	**median**	**1.43**	**2.82**	**0.59**	**5.59**	**1.45**	**0.54**	**0.25**	**1.18**	**0.25**	**0.22**	**0.20**	**0.48**
km^{-2}	90th quantile	2.62	5.11	2.04	8.62	2.88	1.15	0.47	2.95	0.49	0.50	0.44	0.75
n = 871	**mean**	**1.58**	**3.18**	**1.02**	**6.21**	**1.60**	**0.56**	**0.27**	**1.59**	**0.27**	**0.26**	**0.21**	**0.47**
	st. dev.	0.87	2.35	1.89	2.33	1.04	0.55	0.16	1.95	0.21	0.17	0.18	0.22
2000–4000	10th quantile	0.77	1.36	0.16	4.77	0.53	0.02	0.09	0.37	0.09	0.09	0.01	0.21
persons	**median**	**1.41**	**2.86**	**0.56**	**5.62**	**1.45**	**0.55**	**0.25**	**1.25**	**0.25**	**0.21**	**0.20**	**0.48**
km^{-2}	90th quantile	2.68	5.34	2.21	8.47	2.94	1.19	0.50	3.26	0.50	0.50	0.45	0.73
n = 810	**mean**	**1.61**	**3.34**	**1.11**	**6.18**	**1.66**	**0.58**	**0.28**	**1.76**	**0.27**	**0.26**	**0.22**	**0.47**
	st. dev.	0.89	2.52	1.94	1.71	1.11	0.59	0.17	2.33	0.18	0.17	0.19	0.22
4000+	10th quantile	0.75	1.57	0.14	5.42	0.70	0.02	0.11	0.43	0.10	0.07	0.01	0.22
persons	**median**	**1.39**	**3.20**	**0.67**	**5.86**	**1.63**	**0.66**	**0.27**	**1.36**	**0.27**	**0.22**	**0.21**	**0.50**
km^{-2}	90th quantile	2.53	5.77	2.45	7.67	2.94	1.18	0.48	3.29	0.50	0.50	0.42	0.71
n = 489	**mean**	**1.58**	**3.96**	**1.52**	**6.21**	**1.79**	**0.64**	**0.29**	**1.98**	**0.30**	**0.26**	**0.21**	**0.47**
	st. dev.	0.83	5.12	4.73	1.29	1.11	0.55	0.16	3.37	0.36	0.18	0.18	0.22

[a] Concentration values (C_i, C_{iie}, C_o, C_{io}, C_{iip}, C_{ii}) are reported in units of µg m^{-3}. The infiltration factor, F_{inf}, and the fractions of indoor $PM_{2.5}$ of indoor episodic origin, indoor persistent origin, and total indoor origin, that is C_{iie}/C_i, C_{iip}/C_i, and $(C_{iie}+C_{iip})/C_i$, are unitless. The mean of well-behaved particle loss-rate coefficients at a residence ($\bar{\lambda}_{tot}$) is reported in units of h^{-1}.

SI References

1. K. O'Dell *et al.*, Outside in: the relationship between indoor and outdoor particulate air quality during wildfire smoke events in western US cities. *Environ. Res.: Health* **1**, 015003 (2023).
2. L. Wallace, J. Bi, W.R. Ott, J. Sarnat, Y. Liu, Calibration of low-cost PurpleAir outdoor monitors using an improved method of calculating $PM_{2.5}$. *Atmos. Environ.* **256**, 118432 (2021).
3. L. Wallace, T. Zhao, N.E. Klepeis, Calibration of PurpleAir PA-I and PA-II Monitors Using Daily Mean $PM_{2.5}$ Concentrations Measured in California, Washington, and Oregon from 2017 to 2021. *Sensors* **22**, 4741 (2022).
4. L. Morawska *et al.*, Indoor aerosols: from personal exposure to risk assessment. *Indoor Air* **23**, 462–487 (2013).
5. P. deSouza *et al.*, An analysis of degradation in low-cost particulate matter sensors. *Environ. Sci.: Atmos.* **3**, 521–536 (2023).
6. A.L. Holder *et al.*, Field Evaluation of Low-Cost Particulate Matter Sensors for Measuring Wildfire Smoke. *Sensors* **20**, 4796 (2020).
7. Y. Liang *et al.*, Wildfire smoke impacts on indoor air quality assessed using crowdsourced data in California. *Proc. Natl. Acad. Sci. U.S.A.* **118**, e2106478118 (2021).
8. W. Ott, L. Wallace, D. Mage, Predicting Particulate (PM_{10}) Personal Exposure Distributions Using a Random Component Superposition Statistical Model. *J. Air Waste Manage. Assoc.* **50**, 1390–1406 (2000).
9. C. Wu, J.Z. Yu, Evaluation of linear regression techniques for atmospheric applications: the importance of appropriate weighting. *Atmos. Meas. Tech.* **11**, 1233–1250 (2018).
10. B.E. Cummings, A.M. Avery, P.F. DeCarlo, M.S. Waring, Improving Predictions of Indoor Aerosol Concentrations of Outdoor Origin by Considering the Phase Change of Semivolatile Material Driven by Temperature and Mass-Loading Gradients, *Environ. Sci. Technol.* **55**, 9000–9011 (2021).
11. M.M. Lunden *et al.*, The transformation of outdoor ammonium nitrate aerosols in the indoor environment. *Atmos. Environ.* **37**, 5633–5644 (2003).
12. A.M. Avery, M.S. Waring, P.F. DeCarlo, Seasonal variation in aerosol composition and concentration upon transport from the outdoor to indoor environment, *Environ. Sci.: Process. Impacts* **21**, 528–547 (2019).
13. V. Shah *et al.*, Chemical feedbacks weaken the wintertime response of particulate sulfate and nitrate to emissions reductions over the eastern United States. *Proc. Natl. Acad. Sci. U.S.A.* **115**, 8110–8115 (2018).
14. A. van Donkelaar, R.V. Martin, C. Li, R.T. Burnett, Regional Estimates of Chemical Composition of Fine Particulate Matter Using a Combined Geoscience-Statistical Method with Information from Satellites, Models, and Monitors. *Environ. Sci. Technol.* **53**, 2595–2611 (2019).
